# Discovering common pathogenetic processes between COVID-19 and tuberculosis by bioinformatics and system biology approach

**DOI:** 10.3389/fcimb.2023.1280223

**Published:** 2023-12-15

**Authors:** Tengda Huang, Jinyi He, Xinyi Zhou, Hongyuan Pan, Fang He, Ao Du, Bingxuan Yu, Nan Jiang, Xiaoquan Li, Kefei Yuan, Zhen Wang

**Affiliations:** ^1^ Division of Liver Surgery, Department of General Surgery and Laboratory of Liver Surgery, and State Key Laboratory of Biotherapy, West China Hospital, Sichuan University, Chengdu, China; ^2^ Center of Infectious Diseases, West China Hospital, Sichuan University, Chengdu, China

**Keywords:** SARS-CoV-2, tuberculosis, differentially expressed genes, protein-protein interaction (PPI), hub gene, drug molecule

## Abstract

**Introduction:**

The coronavirus disease 2019 (COVID-19) pandemic, stemming from the severe acute respiratory syndrome coronavirus 2 (SARS-CoV-2), has persistently threatened the global health system. Meanwhile, tuberculosis (TB) caused by *Mycobacterium tuberculosis* (*M. tuberculosis*) still continues to be endemic in various regions of the world. There is a certain degree of similarity between the clinical features of COVID-19 and TB, but the underlying common pathogenetic processes between COVID-19 and TB are not well understood.

**Methods:**

To elucidate the common pathogenetic processes between COVID-19 and TB, we implemented bioinformatics and systematic research to obtain shared pathways and molecular biomarkers. Here, the RNA-seq datasets (GSE196822 and GSE126614) are used to extract shared differentially expressed genes (DEGs) of COVID-19 and TB. The common DEGs were used to identify common pathways, hub genes, transcriptional regulatory networks, and potential drugs.

**Results:**

A total of 96 common DEGs were selected for subsequent analyses. Functional enrichment analyses showed that viral genome replication and immune-related pathways collectively contributed to the development and progression of TB and COVID-19. Based on the protein-protein interaction (PPI) network analysis, we identified 10 hub genes, including IFI44L, ISG15, MX1, IFI44, OASL, RSAD2, GBP1, OAS1, IFI6, and HERC5. Subsequently, the transcription factor (TF)–gene interaction and microRNA (miRNA)–gene coregulatory network identified 61 TFs and 29 miRNAs. Notably, we identified 10 potential drugs to treat TB and COVID-19, namely suloctidil, prenylamine, acetohexamide, terfenadine, prochlorperazine, 3′-azido-3′-deoxythymidine, chlorophyllin, etoposide, clioquinol, and propofol.

**Conclusion:**

This research provides novel strategies and valuable references for the treatment of tuberculosis and COVID-19.

## Introduction

Coronavirus disease 2019 (COVID-19), resulting from severe acute respiratory syndrome coronavirus 2 (SARS-CoV-2), is an atypical respiratory disease (Pollard et al., 2020). According to the World Health Organization (WHO), as of January 2023, there have been more than 659 million confirmed cases and over 6.6 million deaths worldwide. The common symptoms of COVID-19 include fever, dyspnea, dizziness, upper airway congestion, dry cough, and sputum production (Jin et al., 2020). Additionally, vomiting, headaches, dizziness, loss of taste and smell, and diarrhea have also been reported (Shi et al., 2020; Spinato et al., 2020). The primary mode of SARS-CoV-2 transmission is through respiratory droplets released when an infected person sneezes or coughs, potentially infecting individuals in close proximity (Umakanthan et al., 2020). SARS-CoV-2 belongs to the β coronaviruses and is composed of four structural proteins: spike (S), nucleocapsid (N), membrane (M), and envelope (E) (Chan et al., 2020). The spike protein plays a critical role in binding to host cell receptors and facilitating the fusion of cellular and viral membranes. Angiotensin-converting enzyme 2 (ACE2) is a pivotal receptor for SARS-CoV-2’s invasion of host cells and is abundantly present in the bladder, heart, lung, kidney, and ileum (Chen et al., 2020; Gheblawi et al., 2020; Zou et al., 2020). Certain preexisting conditions substantially elevate the risk of severe complications and mortality among COVID-19 patients (Huang et al., 2023b; Huang et al., 2023a; Song et al., 2023).

Tuberculosis (TB), caused by *Mycobacterium tuberculosis* (*M. tuberculosis*), is a grave infectious disease that poses a significant threat to global public health due to its high global mortality and morbidity rates (Behr et al., 2021). TB can affect individuals of all age groups and both sexes, with adult men constituting 56% of all TB cases (Chakaya et al., 2021). TB is a severe infectious pulmonary disorder, resulting in pulmonary consolidation, cavitary lesions, and bronchial wall thickening (Deshpande et al., 2020). Furthermore, COVID-19 patients with active pulmonary tuberculosis face a higher risk of mortality due to compromised lung immunity in comparison to patients without tuberculosis (Aggarwal et al., 2021). Additionally, individuals with severe COVID-19 are at greater risk of TB infection compared to those with milder cases (Gao et al., 2021). These studies illustrate that there is a strong interaction and association between TB and COVID-19.

As our understanding of these diseases has deepened, numerous similarities between TB and COVID-19 have been discovered in terms of pathogenesis, clinical symptoms, and sequelae. Within host cells, both *M. tuberculosis* and SARS-CoV-2 can induce proinflammatory cytokines, potentially leading to a cytokine storm if not properly regulated, and they share similar mechanisms for evading the immune system and host cell responses (Zhai et al., 2019; Ragab et al., 2020; Callaway, 2021). Notably, *M. tuberculosis* infection boosts the expression of ACE2, causing significantly severe multi-organ injury (Ziegler et al., 2020). Bacillus Calmette–Guérin (BCG), a weakened live vaccine against *M. tuberculosis*, is helpful in decreasing the proportion of incidence of SARS-CoV-2 IgG seroconversion and clinical symptoms in COVID-19 patients (Benn et al., 2013; Rivas et al., 2021).

**Figure 1:**
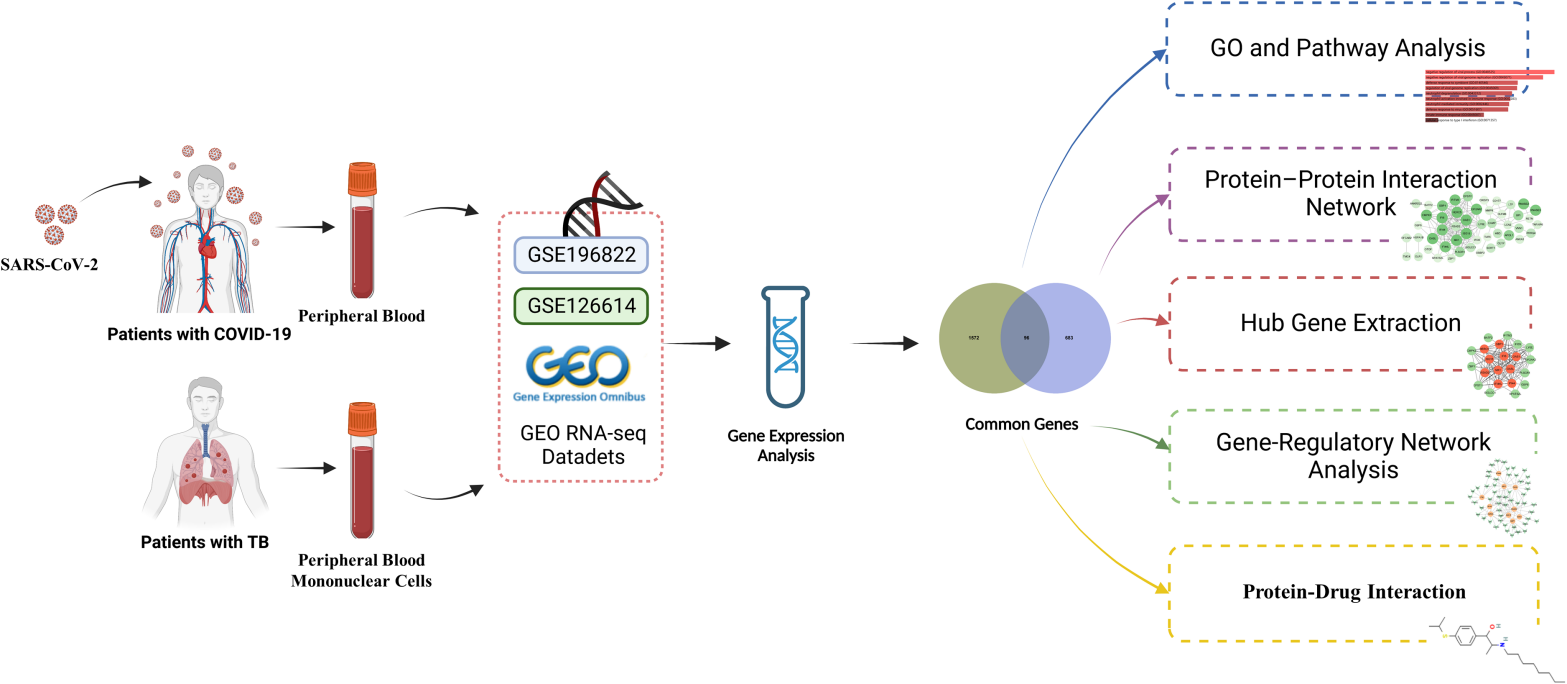
A schematic illustration of the overall general workflow of this study.

Exploring the transcriptional profiles of TB and COVID-19 may provide new insights into the common pathogenesis of the two diseases. The TB datasets (GSE126614) and COVID-19 datasets (GSE196822) were obtained from the Gene Expression Omnibus (GEO) database. Then differentially expressed genes (DEGs) in TB and COVID-19 were filtrated, and their shared DEGs were identified to investigate their functions in these two diseases. In addition, we utilized the common DEGs to establish a protein–protein interaction (PPI) network chart and extracted the hub genes, which are used for the recognition of engaged transcription factors (TF), microRNAs (miRNA), and the prediction of potential drugs. The sequential workflow of the analysis is presented in **Figure 1**.

## Methods

### Data sources

To determine common pathogenetic processes among TB and COVID-19, we used RNA-seq datasets from the GEO database of the National Center for Biotechnology Information (NCBI, https://www.ncbi.nlm.nih.gov/geo/) (Barrett et al., 2013). The GEO accession number of the COVID-19 dataset was GSE196822, which was transcriptional profiling of the peripheral blood of 34 patients with COVID-19 and nine healthy individuals. The COVID-19 dataset was obtained through high-throughput sequencing on the Illumina Hiseq 4000 platform (*Homo sapiens*) for extracting RNA sequences (Huang et al., 2023a; Li et al., 2022). For the TB dataset, we utilized the GEO accession ID of GSE126614 (Del Rosario et al., 2022), which contains the transcriptomic profiles for peripheral blood mononuclear cells from 19 healthy controls and 20 patients with active TB infection. The TB dataset was obtained through high-throughput sequencing on the Illumina HiSeq 2000 system (*Homo sapiens*). **Supplementary Tables S1**, **S2** show the baseline characteristics of samples in COVID-19 and TB.

### Identification of DEGs and common DEGs in COVID-19 and TB

A statistically significant difference between diverse test circumstances at the transcriptional level is generally accepted as the criterion for determining whether the genes are expressed differently (Kvam et al., 2012). The DEGs were detected from the expression read count values by the DEseq2 R package with a Benjamini–Hochberg correction to control the false discovery rate (FDR) (Love et al., 2014). The main role of the analysis is to acquire DEGs for the GSE196822 and GSE126614 datasets. The genes that comply with |log_2_ Fold Change| > 1 and FDR < 0.05 were viewed as significantly DEGs. The mutual DEGs of GSE196822 and GSE126614 were obtained by Jvenn (http://jvenn.toulouse.inra.fr/app/example.html), an online Venn analysis program (Bardou et al., 2014).

### Gene ontology and pathway enrichment analysis

Gene enrichment analysis is a considerable systematic effort to illuminate and categorize shared biological knowledge (Subramanian et al., 2005). EnrichR (https://maayanlab.cloud/enrichr/) is a versatile web-based tool that was used to identify gene ontology functional enrichment (biological processes (BP), molecular function (MF), and cellular component (CC)) and signaling pathway enrichment to clarify potential biological mechanisms of common DEG (Kuleshov et al., 2016). Three databases (Bioplanet, Kyoto Encyclopedia of Genes and Genomes (KEGG), and WikiPathways) were used for pathway enrichment analysis.

### PPI network analysis and hub gene extraction

STRING (version 11.5), a protein interaction database, was utilized to build the PPI network using common DEGs to describe the physical and functional relationship between COVID-19 and TB (Szklarczyk et al., 2019). The medium confidence score set in the analysis was 0.400 to conduct the PPI network. Cytoscape (version 3.9.1) was applied to visualize and process the PPI network (Smoot et al., 2011).

### Hub gene extraction

The PPI network covers edges, nodes, and their links. In this network, the most prominent nodes are supposed to be hub genes. Cytohubba (http://apps.cytoscape.org/apps/cytohubba), a remarkable plug-in in Cytoscape, is used for analyzing nodes and the relationships between them in the PPI network (Ma et al., 2021). Applying the Maximal Clique Centrality (MCC) method of Cytohubba, we confirmed the top 10 genes within the PPI network as the hub genes.

### Gene-regulatory network analysis

Transcription factors are proteins that can specifically identify the corresponding genes and control the transcription rate (Rustad et al., 2014). The gene–TF interaction network was conducted by NetworkAnalyst (http://www.networkanalyst.ca) (Zhou et al., 2019). The topologically credible TFs within the network that were inclined to bind to specific hub genes were from the JASPAR database. JASPAR is an open-access database that contains TF profiles from six taxonomic groups (Fornes et al., 2020). Moreover, miRNAs targeting gene interaction were used to identify miRNAs that have the potential to regulate the hub genes at the post-transcriptional level. MiRTarBase is one of the most known gene–miRNA interplay repositories (Huang et al., 2020). From the miRNA–gene interaction via NetworkAnalyst, we retrieved the miRNAs that can interplay with hub genes concentrated on topological analysis from the miRTarBase database (v 8.0). In Cytoscape, the gene–TF and the gene–miRNA interaction networks were visualized.

### Identification of candidate drugs

The identification of drug molecules is one of the most crucial parts of the research. Based on the hub genes of TB and COVID-19, the drug molecule was discovered using the Drug Signatures Database (DSigDB) via EnrichR. DSigDB is a data management repository for recognizing the chemical compounds of the medicine that correspond with the genes (Yoo et al., 2015). The drug function of EnrichR provides easy access to the DSigDB database.

## Results

### Identification of DEGs and shared DEGs between COVID-19 and TB

**Figure 2:**
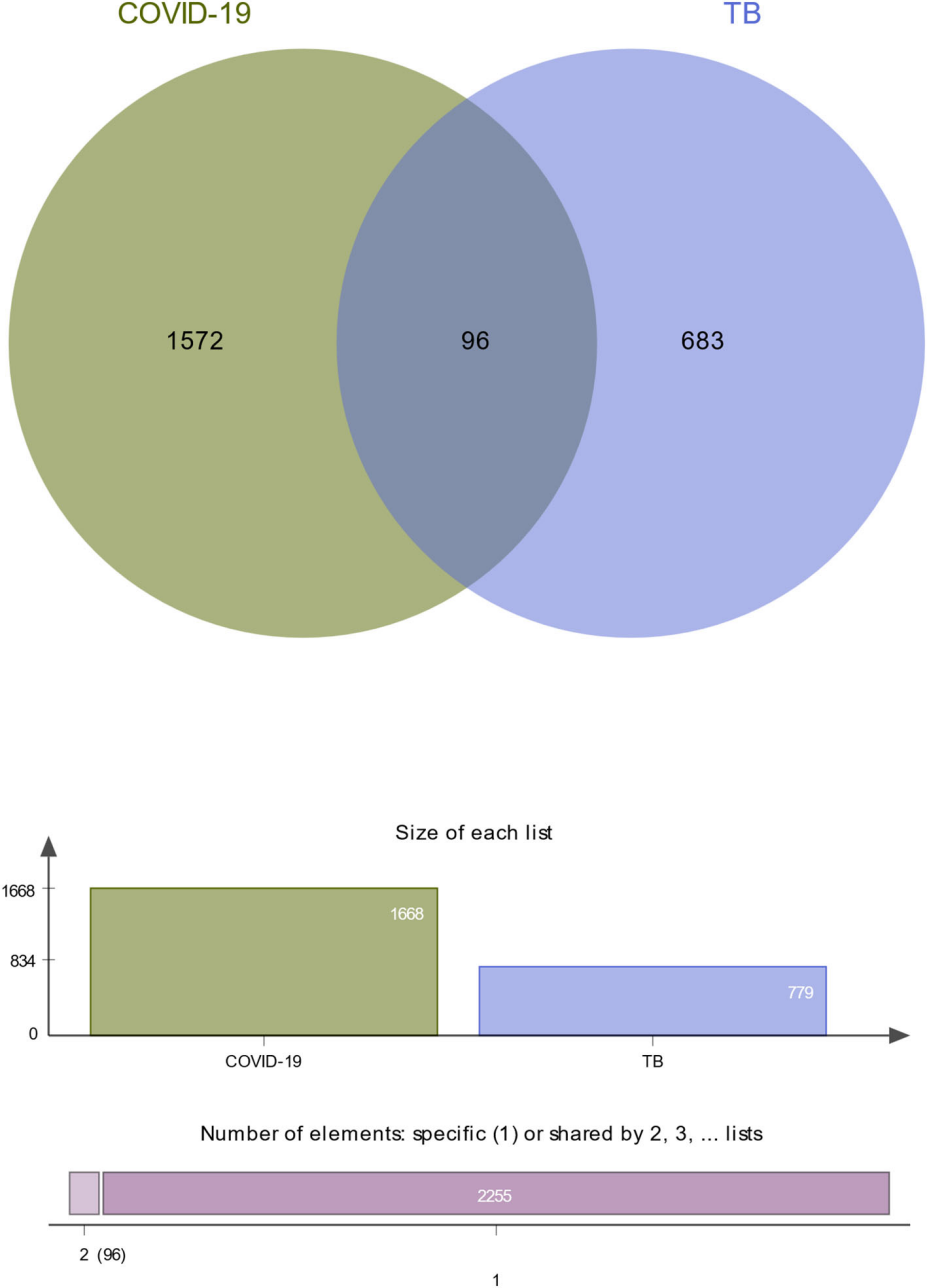
The study incorporates TB (GSE126614) and COVID-19 (GSE196822). The Venn diagram revealed 96 common DEGs for TB and COVID-19.

To investigate the common pathogenetic processes between COVID-19 and TB, we filtrated the DEGs from transcriptional datasets and identified the common DEGs that cause COVID-19 and TB. From the assessment of the COVID-19 dataset (GES196822), there are 1,668 DEGs, including 839 upregulated DEGs and 829 downregulated DEGs (**Supplementary Table S3**). Similarly, based on RNA-seq profiling of patients with TB (GSE126614), we identified 779 DEGs, where 470 DEGs were upregulated and 309 DEGs were downregulated (**Supplementary Table S4**). The summarized information on DEGs for COVID-19 and TB is listed in **Table 1**. Moreover, there are 96 shared DEGs identified from the COVID-19 and TB datasets by the accomplishment of the cross-comparison evaluation on Jvenn (**Figure 2**). These results reveal that the 96 common genes screened in this study mediated the regulation of COVID-19 and TB, suggesting that there are some mechanismal commonalities and common pathogenetic processes between COVID-19 and TB.

**Table 1:** Overview of the datasets in this analysis.

Disease name	GEO accession	GEO platform	Total DEG count	Upmodulated DEG count	Downmodulated DEG count
COVID-19	GES196822	GPL20301	1,668	839	829
TB	GSE126614	GPL11154	779	470	309

### Analyses of gene ontology and pathway enrichment

**Figure 3:**
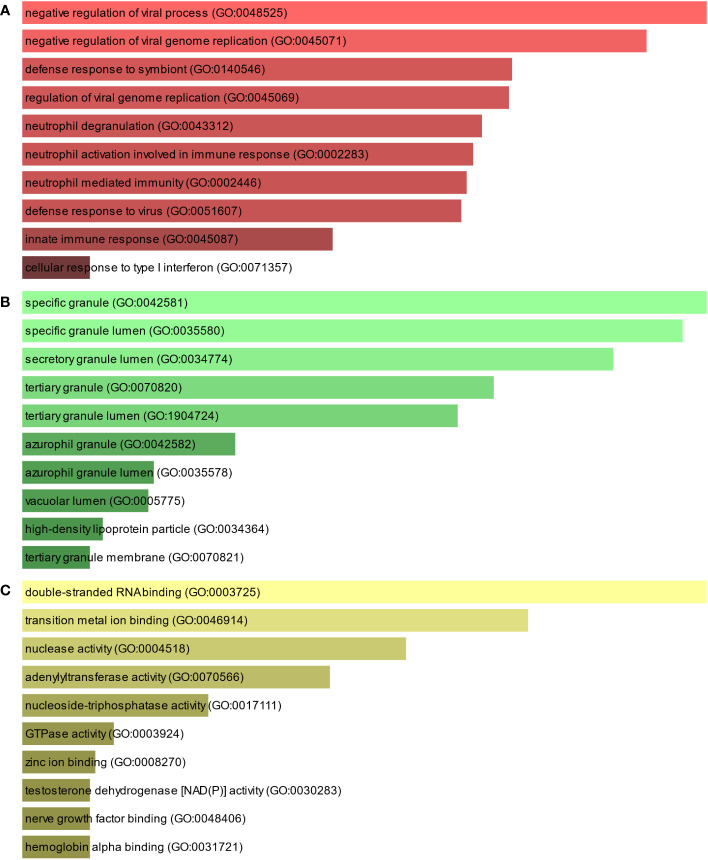
The bar chart of the GO assessment of the shared DEGs between TB and COVID-19. **(A)** Biological processes, **(B)** Cellular components, and **(C)** Molecular function.

To further understand the biological significance and the common signaling pathways of the dataset, we used the common DEGs to implement the GO enrichment approach and pathway enrichment method through EnrichR. For gene ontology analysis, the top 10 terms related to the biological process, molecular function, and cellular component categories are summarized in **Table 2**. The bar graph in **Figure 3** indicates the comprehensive ontological analysis for each category. Notably, viral genome replication and immune-related pathways are significantly enriched, including negative regulation of viral process (GO:0048525), negative regulation of viral genome replication (GO:0045071), regulation of viral genome replication (GO:0045069), neutrophil activation involved in immune response (GO:0002283), neutrophil-mediated immunity (GO:0002446), defense response to viruses (GO:0051607), and innate immune response (GO:0045087).

**Table 2:** Ontological analysis of common DEGs between TB and COVID-19.

Category	GO ID	Term	*p*-value	Genes
GO biological process	GO:0048525	Negative regulation of the viral process	2.96*E*−14	IFITM3; PARP10; PLSCR1; RSAD2; OAS1; MX1; EIF2AK2; ISG15; PML; OASL; LTF
	GO:0045071	Negative regulation of viral genome replication	8.07*E*−14	IFITM3; PARP10; PLSCR1; RSAD2; OAS1; MX1; EIF2AK2; ISG15; OASL; LTF
	GO:0140546	Defense response to symbiont	7.55*E*−13	IFITM3; ZBP1; PLSCR1; RSAD2; OAS1; MX1; IFI6; EIF2AK2; ISG15; RNASE2; IFI44L; OASL
	GO:0045069	Regulation of viral genome replication	7.94*E*−13	IFITM3; PARP10; PLSCR1; RSAD2; OAS1; MX1; EIF2AK2; ISG15; OASL; LTF
	GO:0043312	Neutrophil degranulation	1.25*E*−12	TNFAIP6; ANXA3; CRISP3; RNASE3; RETN; OLFM4; MMP8; RNASE2; VNN1; LCN2; OLR1; BPI; S100P; GYG1; CAMP; HSPA1B; CD177; LTF; SIGLEC5
	GO:0002283	Neutrophil activation is involved in the immune response	1.44*E*−12	TNFAIP6; ANXA3; CRISP3; RNASE3; RETN; OLFM4; MMP8; RNASE2; VNN1; LCN2; OLR1; BPI; S100P; GYG1; CAMP; HSPA1B; CD177; LTF; SIGLEC5
	GO:0002446	Neutrophil-mediated immunity	1.60*E*−12	TNFAIP6; ANXA3; CRISP3; RNASE3; RETN; OLFM4; MMP8; RNASE2; VNN1; LCN2; OLR1; BPI; S100P; GYG1; CAMP; HSPA1B; CD177; LTF; SIGLEC5
	GO:0051607	Defense response to virus	1.75*E*−12	IFITM3; ZBP1; PLSCR1; RSAD2; OAS1; MX1; IFI6; EIF2AK2; ISG15; RNASE2; IFI44L; OASL
	GO:0045087	Innate immune response	1.49*E*−11	IFITM3; CRISP3; MX1; IFI6; ISG15; RNASE3; RNASE2; PML; VNN1; OAS1; LCN2; BPI; APOL1; CAMP; LTF
	GO:0071357	Cellular response to type I interferon	8.44*E*−10	IFITM3; RSAD2; OAS1; MX1; IFI6; ISG15; IFI35; OASL
GO cellular component	GO:0042581	Specific granule	2.98*E*−10	ANXA3; CRISP3; LCN2; OLR1; BPI; RETN; OLFM4; MMP8; CAMP; CD177; LTF
	GO:0035580	Specific granule lumen	5.72*E*−10	CRISP3; LCN2; BPI; RETN; MMP8; OLFM4; CAMP; LTF
	GO:0034774	Secretory granule lumen	3.72*E*−09	CRISP3; RNASE3; RETN; OLFM4; MMP8; RNASE2; LCN2; SERPING1; BPI; S100P; GYG1; CAMP; LTF
	GO:0070820	Tertiary granule	9.47*E*−08	TNFAIP6; CRISP3; OLR1; OLFM4; MMP8; CAMP; CD177; LTF; SIGLEC5
	GO:1904724	Tertiary granule lumen	2.50*E*−07	TNFAIP6; CRISP3; MMP8; OLFM4; CAMP; LTF
	GO:0042582	Azurophil granule	1.03*E*−04	VNN1; BPI; RNASE3; RETN; OLFM4; RNASE2
	GO:0035578	Azurophil granule lumen	9.29*E*−04	BPI; RNASE3; RETN; RNASE2
	GO:0005775	Vacuolar lumen	1.08*E*−03	BPI; RNASE3; RETN; RNASE2; GYG1
	GO:0034364	High-density lipoprotein particle	3.70*E*−03	CETP; APOL1
	GO:0070821	Tertiary granule membrane	5.23*E*−03	OLR1; CD177; SIGLEC5
GO molecular function	GO:0003725	Double-stranded RNA binding	4.21*E*−04	ZBP1; OAS1; EIF2AK2; OASL
	GO:0046914	Transition metal ion binding	1.36*E*−03	RPH3A; MT2A; PLSCR1; LCN2; TIMM10; MMP8; GYG1; LTF
	GO:0004518	Nuclease activity	3.01*E*−03	PLSCR1; RNASE3; RNASE2
	GO:0070566	Adenylyltransferase activity	4.95*E*−03	OAS1; OASL
	GO:0017111	Nucleoside-triphosphatase activity	1.10*E*−02	GBP6; MX1; RHOH; GBP1; HSPA1B
	GO:0003924	GTPase activity	2.03*E*−02	GBP6; MX1; RHOH; GBP1
	GO:0008270	Zinc ion binding	2.30*E*−02	RPH3A; MT2A; PLSCR1; TIMM10; MMP8
	GO:0030283	Testosterone dehydrogenase [NAD(P)] activity	2.38*E*−02	DHRS9
	GO:0048406	Nerve growth factor binding	2.38*E*−02	SORT1
	GO:0031721	Hemoglobin alpha binding	2.38*E*−02	HBD

**Figure 4:**
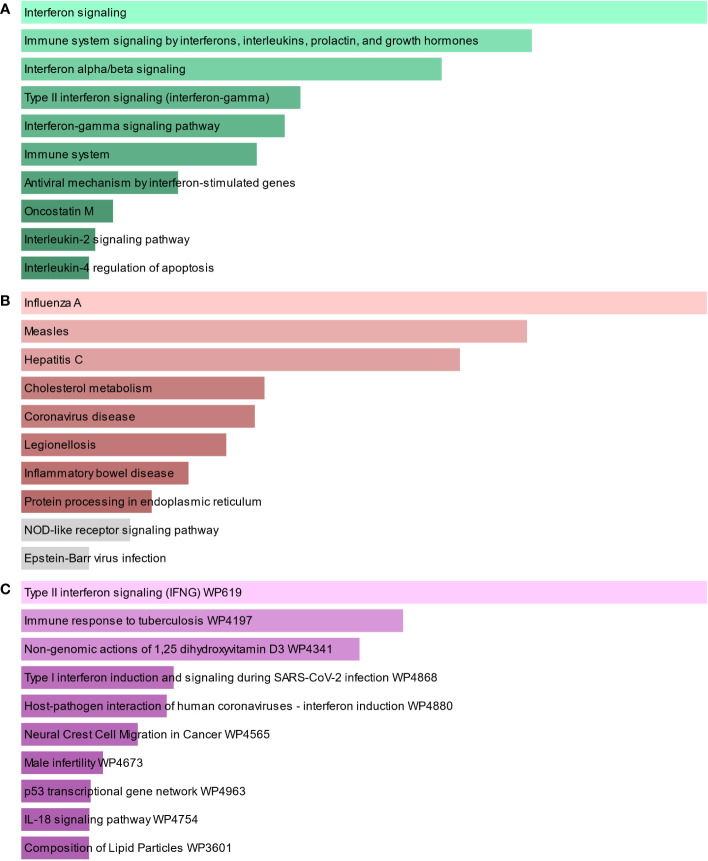
The bar graphs of the pathway enrichment of the shared DEGs between TB and COVID-19. **(A)** Bioplanet, **(B)** KEGG, and **(C)** WikiPathways.

To find the most significant pathways of the mutual DEGs, three global databases were utilized, including Bioplanet, KEGG, and WikiPathways. The identified top 10 pathways in the three databases are outlined in **Table 3**. Moreover, the pathway enrichment study is described in the bar graph in **Figure 4**. The Bioplanet enrichment analysis showed that the common DEGs are mainly involved in the regulation of immune-related pathways, including interferon signaling, interferon alpha/beta signaling, type II interferon signaling (interferon-gamma), interferon-gamma signaling pathway, immune system, antiviral mechanism by interferon-stimulated genes, and interleukin-2 signaling pathway. Furthermore, the KEGG analysis showed the shared DEGs may influence the progression of a variety of infectious diseases, including coronavirus disease, influenza A, measles, hepatitis C, and Epstein–Barr virus infection. Most important of all, the WikiPathways analysis revealed mutual DEGs significantly enriched in the immune response to tuberculosis (WP4197), type I interferon induction and signaling during SARS-CoV-2 infection (WP4868), and host–pathogen interaction of human coronaviruses—interferon induction (WP4880). These results strongly suggest that these mutual DEGs are involved in the occurrence and development of these two infectious diseases through viral genome replication and immune-related pathways.

**Table 3:** Pathway enrichment analysis of common DEGs between TB and COVID-19.

Category	Pathways	*p*-value	Genes
Bioplanet	Interferon signaling	1.43*E*−12	IFITM3; GBP6; MX1; IFI6; EIF2AK2; ISG15; IFI35; PML; OASL; HERC5; MT2A; OAS1; GBP1
	Immune system signaling by interferons, interleukins, prolactin, and growth hormones	8.61*E*−10	IFITM3; GBP6; MX1; IFI6; EIF2AK2; ISG15; IFI35; PML; OASL; HERC5; MT2A; OAS1; GBP1
	Interferon alpha/beta signaling	2.34*E*−08	IFITM3; OAS1; MX1; IFI6; ISG15; IFI35; OASL
	Type II interferon signaling (interferon-gamma)	4.10*E*−06	OAS1; IFI6; EIF2AK2; ISG15; GBP1
	Interferon-gamma signaling pathway	7.26*E*−06	GBP6; MT2A; OAS1; GBP1; PML; OASL
	Immune system	2.02*E*−05	IFITM3; ZBP1; GBP6; MX1 IFI6; EIF2AK2; ISG15; IFI35; PML; OASL; HERC5; MT2A; OAS1; FBXO6; TLR5; GBP1
	Antiviral mechanism by interferon-stimulated genes	3.59*E*−04	HERC5; MX1; EIF2AK2; ISG15
	Oncostatin M	3.87*E*−03	MT2A; OAS1; ANXA3; LCN2; S100P; CAMP
	Interleukin-2 signaling pathway	7.43*E*−03	IFITM3; MT2A; SMARCD3; IL24; MX1; TBC1D8; IFI44; GBP1; IL18R1; LY6E
KEGG	Influenza A	1.44*E*−03	RSAD2; OAS1; MX1; EIF2AK2; PML
	Measles	4.53*E*−03	OAS1; MX1; EIF2AK2; HSPA1B
	Hepatitis C	6.94*E*−03	RSAD2; OAS1; MX1; EIF2AK2
	Cholesterol metabolism	2.41*E*−02	CETP; SORT1
	Coronavirus disease	2.56*E*−02	OAS1; MX1; EIF2AK2; ISG15
	Legionellosis	3.07*E*−02	TLR5; HSPA1B
	Inflammatory bowel disease	3.90*E*−02	TLR5; IL18R1
	Protein processing in the endoplasmic reticulum	4.93*E*−02	EIF2AK2; FBXO6; HSPA1B
	NOD-like receptor signaling pathway	5.66*E*−02	OAS1; GBP1; CAMP
	Epstein–Barr virus infection	7.34*E*−02	OAS1; EIF2AK2; ISG15
WikiPathways	Type II interferon signaling (IFNG) WP619	8.85*E*−07	OAS1; IFI6; EIF2AK2; ISG15; GBP1
	Immune response to tuberculosis WP4197	1.77*E*−04	OAS1; MX1; IFI35
	Nongenomic actions of 1,25-dihydroxyvitamin D3 WP4341	3.79*E*−04	RSAD2; ISG15; IFI44L; CAMP
	Type I interferon induction and signaling during SARS-CoV-2 infection WP4868	9.68*E*−03	OAS1; EIF2AK2
	Host–pathogen interaction of human coronaviruses-interferon induction WP4880	1.09*E*−02	OAS1; EIF2AK2
	Neural crest cell migration in cancer WP4565	1.81*E*−02	SORT1; MMP8
	Male infertility WP4673	3.33*E*−02	EPSTI1; BRCA2; LTF
	p53 transcriptional gene network WP4963	4.12*E*−02	ISG15; PML
	IL-18 signaling pathway WP4754	4.21*E*−02	CETP; NRN1; MMP8; IL18R1
	Composition of lipid particles WP3601	4.24*E*−02	CETP

### Protein–protein interaction analysis and hub gene extraction

**Figure 5:**
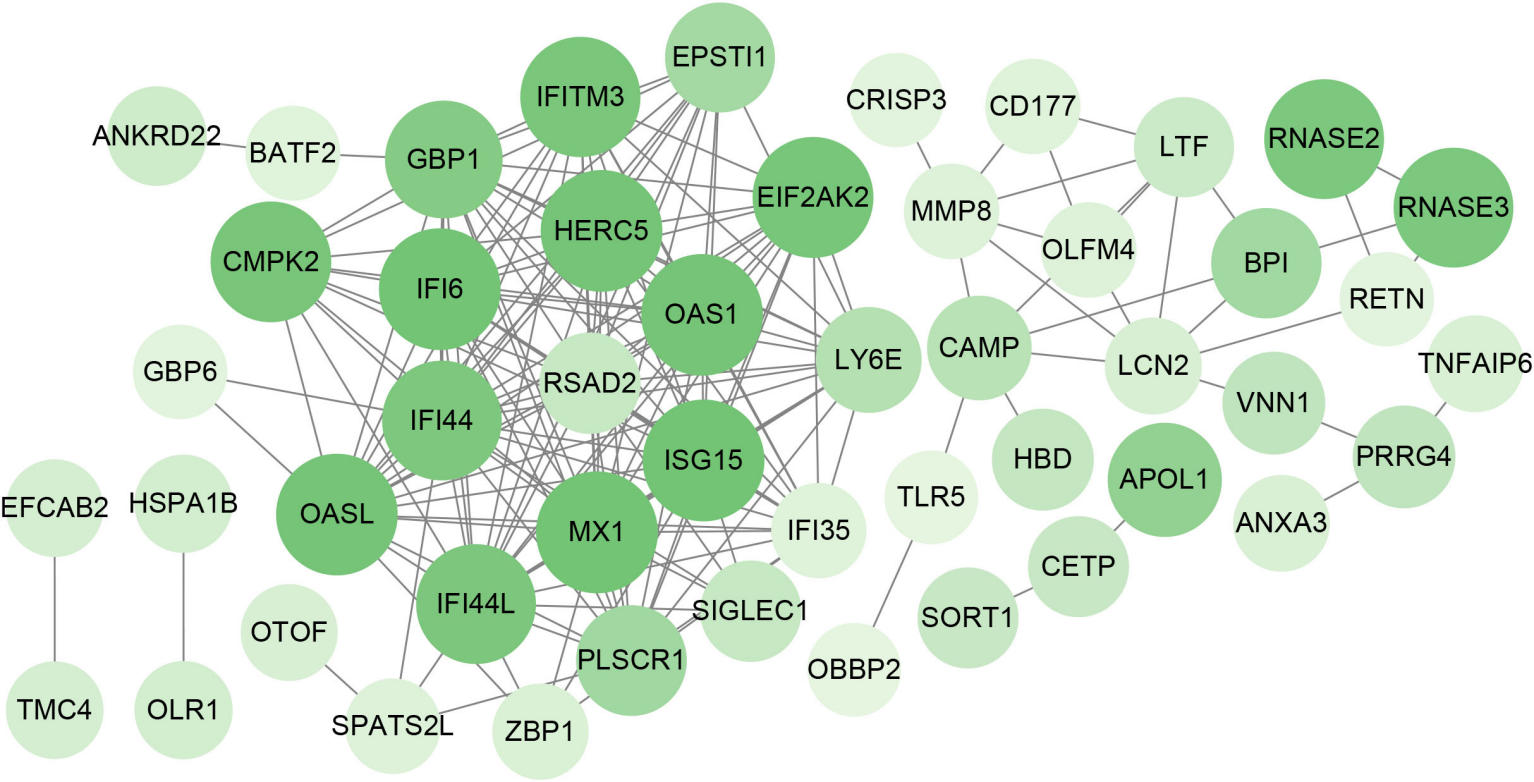
PPI network of the mutual DEGs between COVID-19 and TB. The nodes and the edges of the figure represent DEGs and the interactions between the nodes, respectively. The PPI network contains 176 edges and 49 nodes.

**Figure 6:**
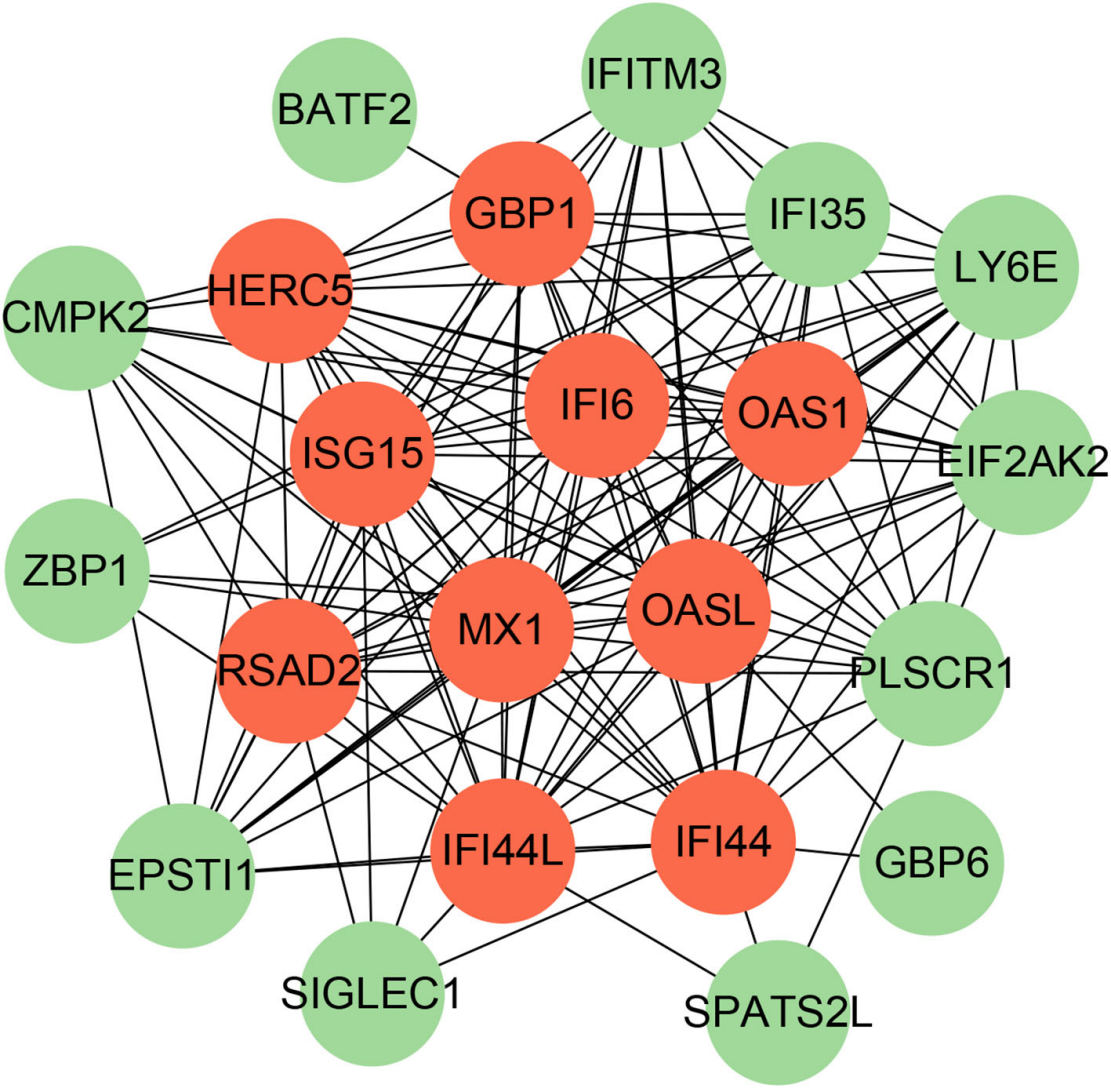
The PPI network from all the shared DEGs is constructed by the Cytohubba plugin in Cytosacpe. Red nodes present the selected top 10 hub genes. The network has 22 nodes and 142 edges.

To identify the interplay and adhesion routes of common DEGs, we analyzed the PPI network constructed from STRING and visualized it in Cytoscape. **Figure 5** demonstrates the PPI network of common DEGs, which consists of 176 edges and 49 nodes. As shown, the size and color depth of the circles indicated the degree of intercorrelation of the proteins, and the most prominent nodes are considered the hub genes. From the PPI network analysis utilizing the Cytohubba plugin, 10 hub genes were selected. **Figure 6** shows the submodule network of hub-gene connections that consists of 22 nodes and 142 edges. These hub genes comprised IFI44L, ISG15, MX1, IFI44, OASL, RSAD2, GBP1, OAS1, IFI6, and HERC5, which could be potential biomarkers for common pathogenetic processes between COVID-19 and TB and may accelerate the development of novel therapeutic strategies.

### Determination of regulatory networks at the transcriptional level

**Figure 7:**
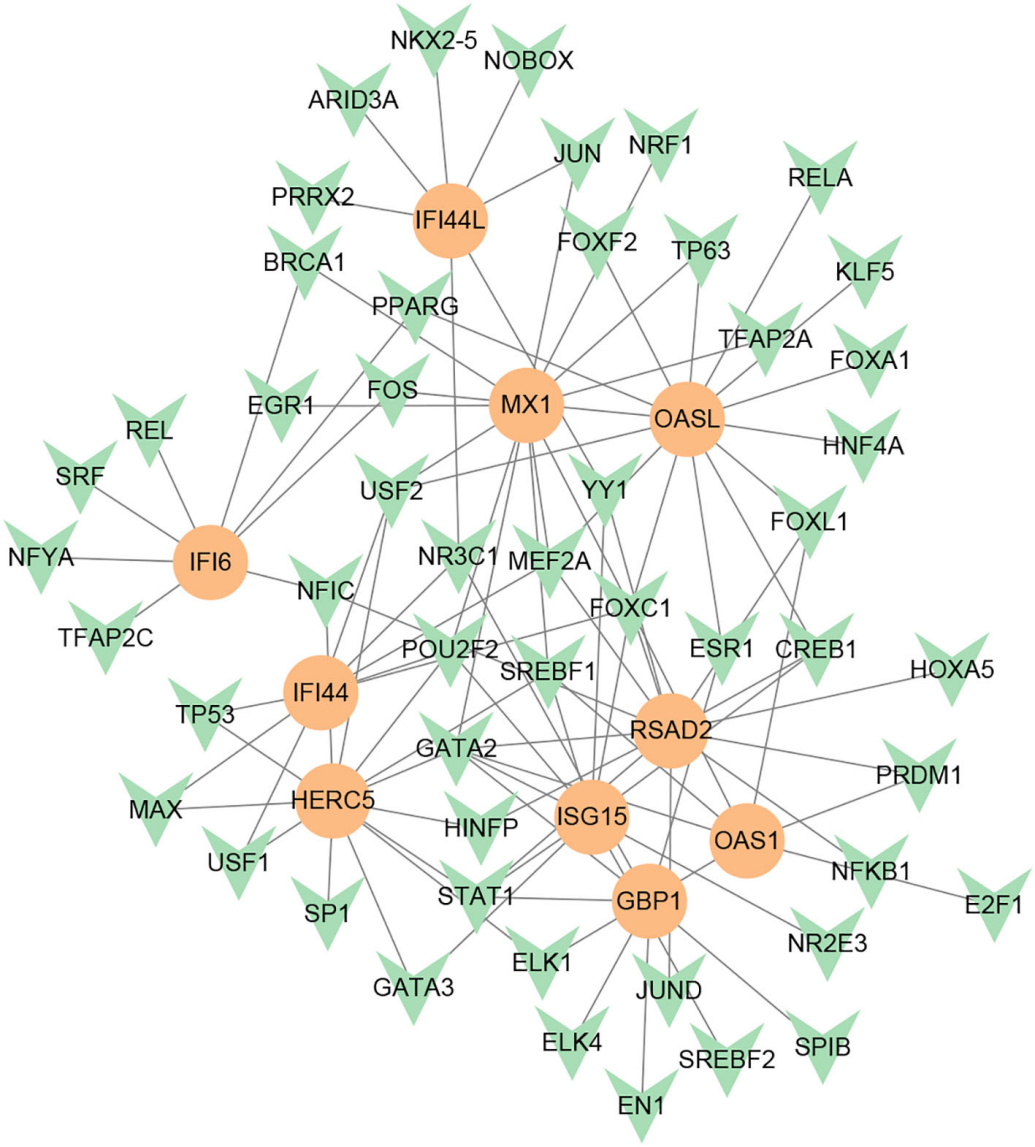
DEG-TF interaction network created by the NetworkAnalyst. The orange nodes represent gene symbols interacting with TFs, while the herringbone nodes represent TFs.

**Figure 8:**
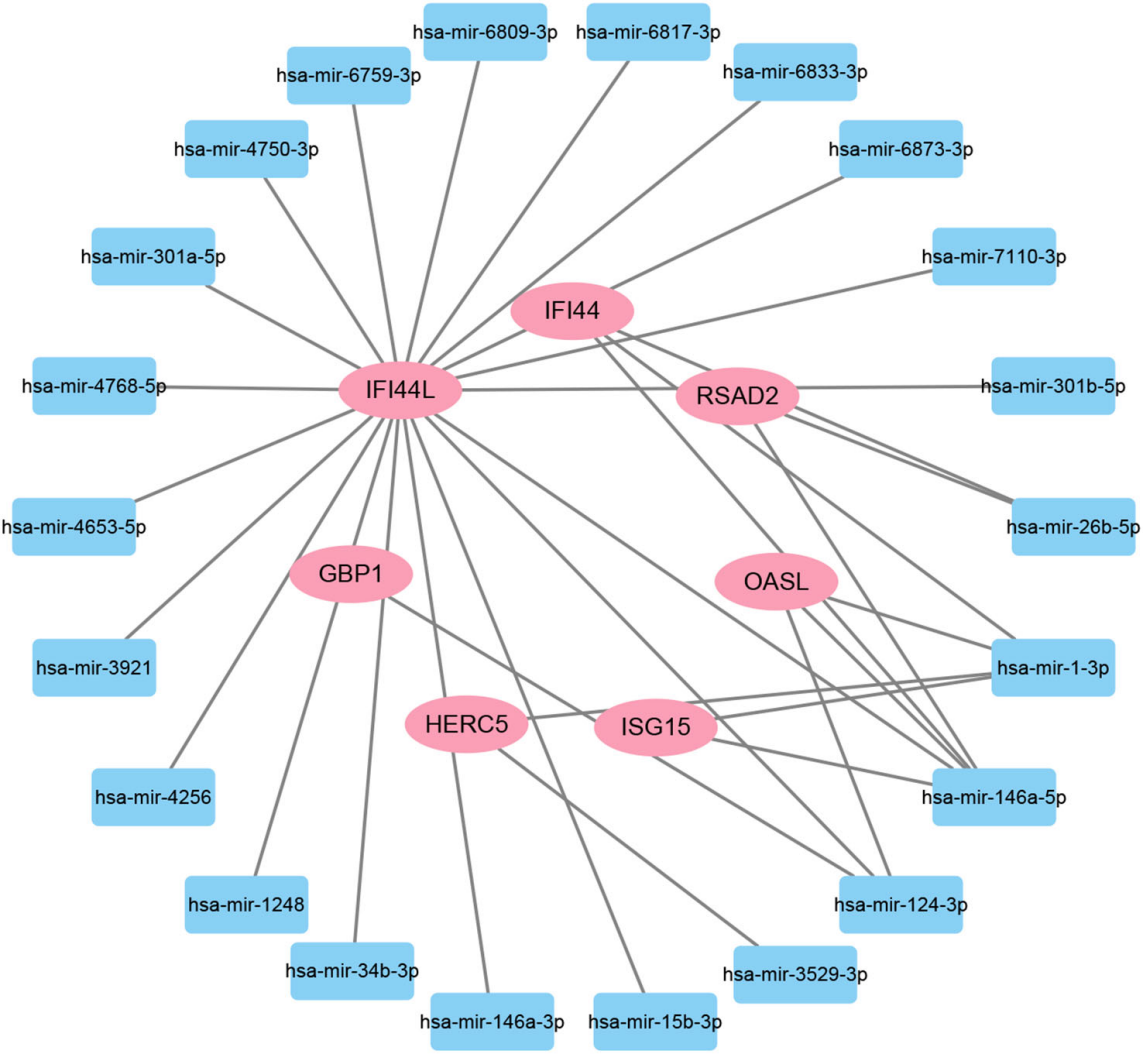
The regulatory interaction network of DEG-miRNAs. MiRNAs are presented by the square node, and gene symbols interacting with miRNAs are in an oval shape.

To investigate the transcriptional regulation of hub genes, a network-based technique was utilized to decipher the regulatory TFs and miRNAs. **Figure 7** shows TF regulators interplay with the hub genes, which have 61 nodes and 99 edges. Moreover, **Figure 8** depicts the interactions of miRNAs and hub genes, consisting of 29 nodes and 32 edges. From TF-gene and miRNA-gene interaction networks, 61 TFs such as GATA2, FOXC1, USF2, MEF2A, STAT1, SREBF1, POU2F2, and 29 miRNAs such as hsa-mir-26b-5p, hsa-mir-15b-3p, hsa-mir-146a-3p, hsa-mir-34b-3p, hsa-mir-1248, hsa-mir-4256, hsa-mir-3921, and hsa-mir-4653-5p, are discovered.

### Identification of candidate drugs

The protein–drug interaction analyses may be useful in drug discovery (Al-Mustanjid et al., 2020). Protein–drug interaction is vital to understanding the function of proteins and discovering advancing drugs. Ten possible drug molecules were predicted using EnrichR based on transcriptome characteristics from the DSigDB database, including suloctidil, prenylamine, acetohexamide, terfenadine, prochlorperazine, 3′-azido-3′-deoxythymidine, chlorophyllin, etoposide, clioquinol, and propofol. The 10 potential medications are extracted in accordance with their *p*-value. **Table 4** depicts the potential drugs in the DSigDB database for hub genes. These potential drugs are recommended for the hub genes, a common compound used to treat two diseases.

**Table 4:** Potential drugs for COVID-19 and TB.

Name	*p-*value	Chemical formula	Structure
Suloctidil HL60 UP	2.19*E*−22	C_20_H_35_NOS	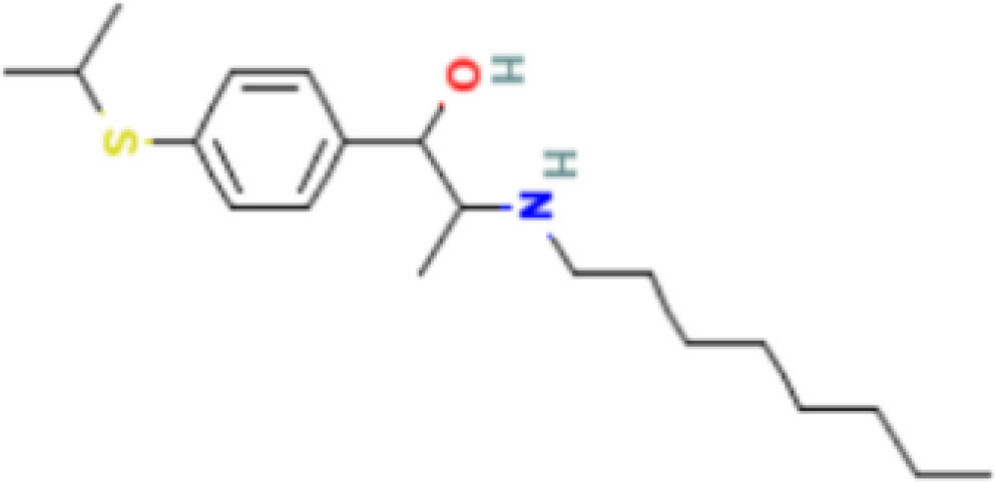
prenylamine HL60 UP	3.45*E*−21	C_24_H_27_N	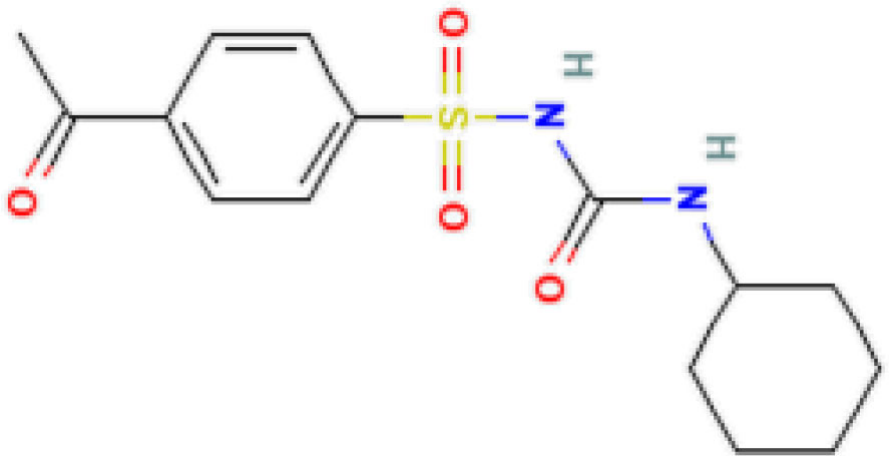
Acetohexamide PC3 UP	1.15*E*−19	C_15_H_20_N_2_O_4_S	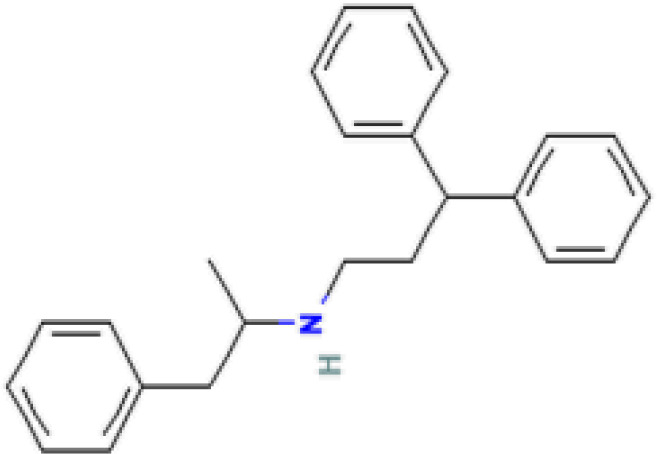
Terfenadine HL60 UP	1.17*E*−13	C_32_H_41_NO_2_	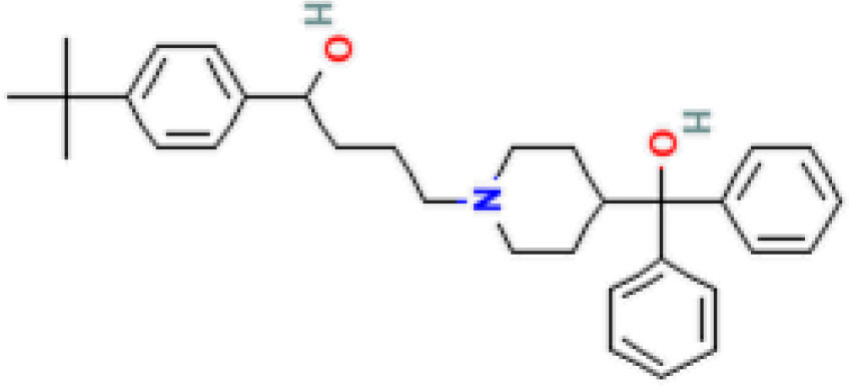
Prochlorperazine MCF7 UP	4.28*E*−11	C_20_H_24_ClN_3_S	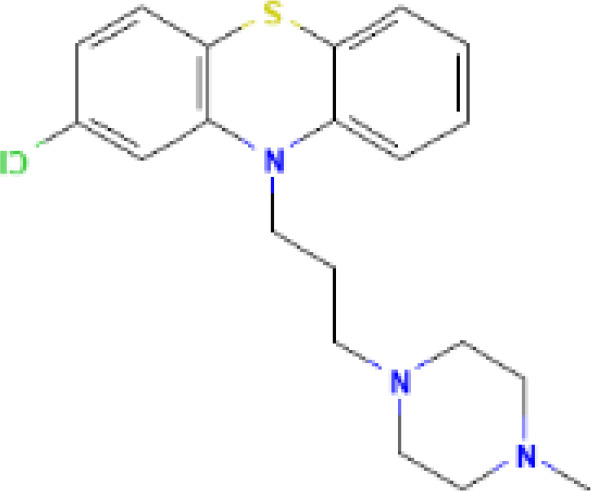
3′-Azido-3′-deoxythymidine CTD 00007047	8.64*E*−11	C_10_H_13_N_5_O_4_	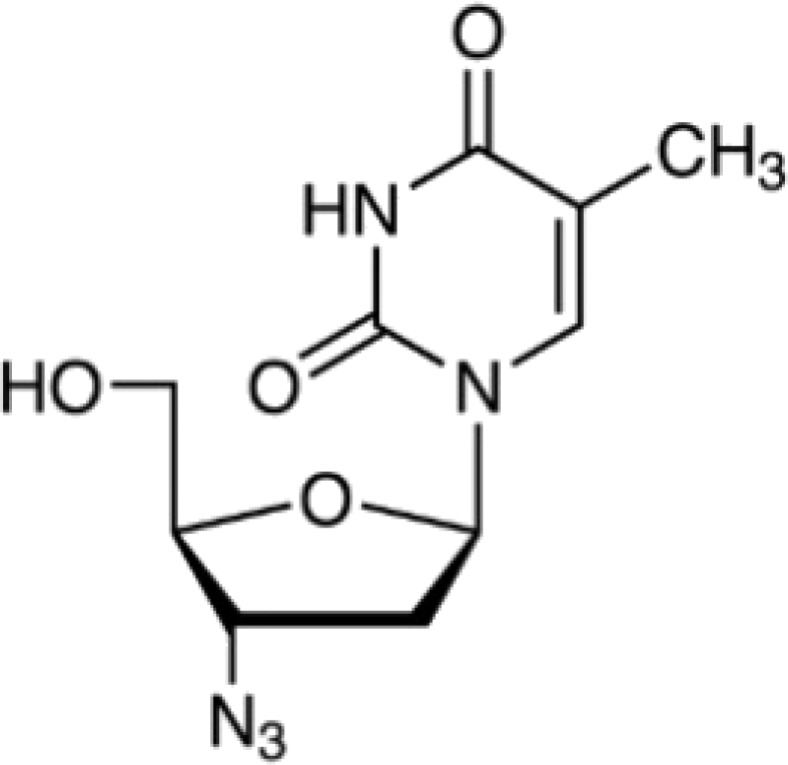
Chlorophyllin CTD 00000324	8.57*E*−10	C_34_H_34_MgN_4_O_6_	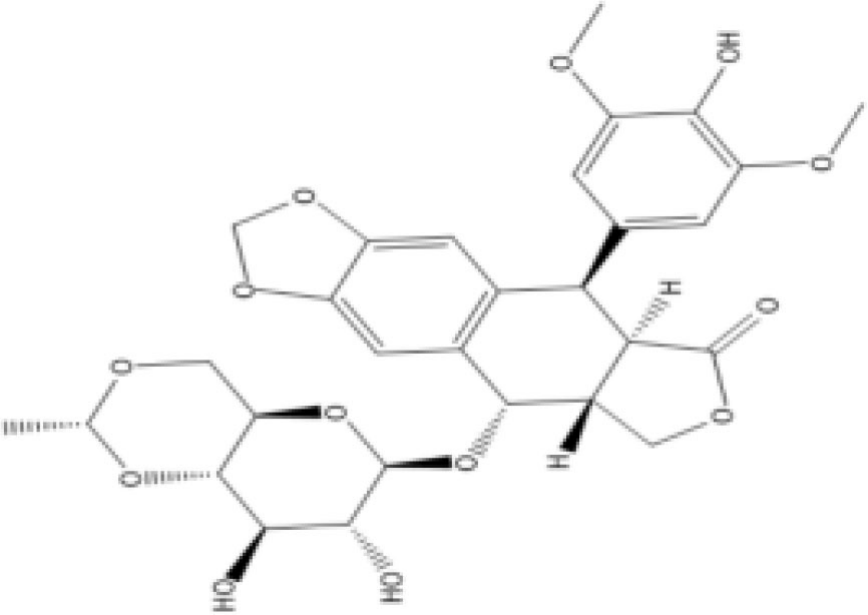
Etoposide HL60 UP	4.90*E*−09	C_29_H_32_O_13_	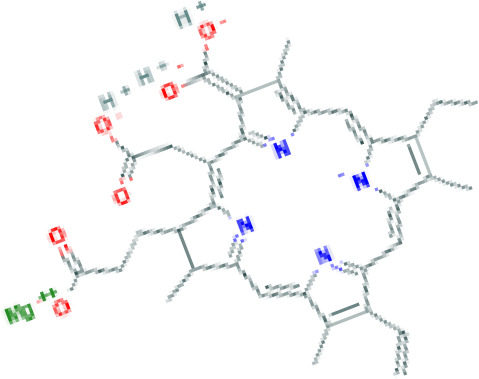
Clioquinol PC3 UP	6.60*E*−09	C_9_H_5_ClINO	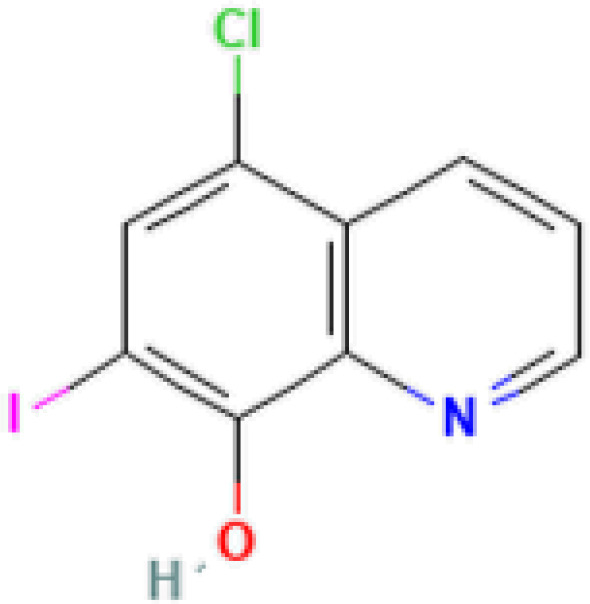
Propofol MCF7 UP	9.84*E*−09	C_12_H_18_O	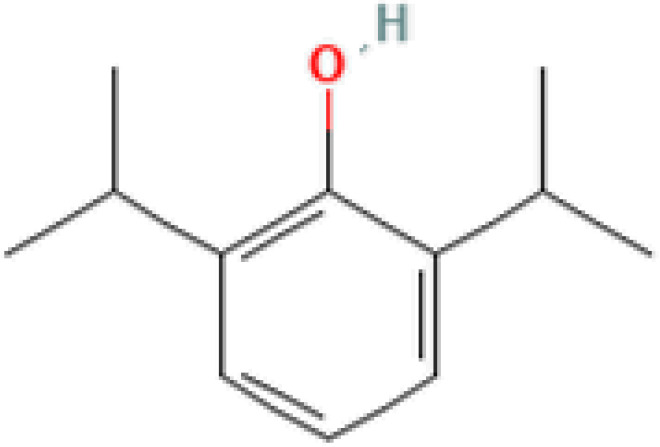

## Discussion

Due to the high incidence and mortality of TB and COVID-19, the main target organ of both is the lung. More importantly, some research recently indicated that there is a strong association between the onset and exacerbation of TB and COVID-19, and likewise, TB is a risk factor for COVID-19 (Fonseca et al., 2021; Gao et al., 2021; Visca et al., 2021). Therefore, it is crucial to explore the common pathogenesis, interaction, and connection between TB and COVID-19.

The enrichment analysis of gene ontology and pathways can help us understand the function and regulation mechanisms of genes in different physiological states. In this study, first of all, 96 common DEGs were identified through the differential analysis of gene expression abundance in transcriptional profiles and the analysis of Venn diagrams, which indicated that there is a certain degree of correlation and similarity between tuberculosis and COVID-19 in their pathogenesis. Subsequently, the functional enrichment analysis of common DEGs was carried out. The results of enrichment analysis showed that these common DEGs were mainly involved in viral genome replication and immune-related pathways. In coronaviruses, the viral genome replication that evades the immunity system may contribute to the viral process (Perlman and Netland, 2009). The negative regulation of viral genome replication is closely tied to the interferon response pathway, and interferon-γ (IFN-γ) is a genome replication negative regulator of SARS-COV-2 (Bhat et al., 2018; Trugilho et al., 2022). SARS-CoV-2 may trigger aggressive proinflammatory reactions in infected cells, including IL-2, IL1-β, IL-4, IL-6, IL-10, IFN-γ, and tumor necrosis factor-α (TNF-α) (Guan et al., 2020). Furthermore, T-cell depletion caused by *M. tuberculosis* affects host immunity, which increases the body’s susceptibility to airborne pathogens, making the host more susceptible to COVID-19 infection (Rahman et al., 2009; Tadolini et al., 2020; Mousquer et al., 2021). In addition, elevated levels of the proinflammatory cytokines IL-6 and TNF-α in COVID-19 patients cause damage to the lymphatic system, resulting in immunosuppression, which contributes to the progression of TB (Tan et al., 2020; Hildebrand et al., 2022).

The shared DEGs are utilized to construct the PPI network, in which the hub gene is the most significant regulator in the common pathogenetic processes of TB and COVID-19. IFI44L is a potential target for reducing viral replication (Dediego et al., 2019). IFI44L promotes positive regulation and eliminates *M. tuberculosis* from human macrophages, highlighting its potential as a therapeutic target against *M. tuberculosis* infection (Jiang et al., 2021; Deng et al., 2023). IFI44 and IFI44L are antiproliferative factors that independently limit respiratory syncytial virus (RSV) infection (Busse et al., 2020). The expression levels of IFI44L and antiviral genes exhibit alterations during SARS-CoV-2 infection in various human cells, such as liver, respiratory epithelial, and stomach cells (Geerling et al., 2022). IFI44 is situated on human chromosome 1p31.1 and is part of the interferon-stimulated gene (ISG) family, which has a crucial function in regulating immunity and recognizing tumor cells (Lukhele et al., 2019; Boutin et al., 2021; Li et al., 2021a; Li et al., 2021b). IFI44 negatively regulates the IFN signaling pathway, promotes viral replication and bacterial proliferation, and is a crucial molecular target for immune evasion by SARS-CoV-2 (Zheng et al., 2022). Although ISG15 antiviral does not directly hinder the viral life cycle, it restricts viral transmission by joining the host response to modify the immune-metabolic network and restrain the availability of resources for viral amplification (Raso et al., 2020; Gold et al., 2022; Munnur et al., 2022). ISG15 can collaborate with IL-12 to stimulate T and NK cells to produce IFN-γ, which regulates *Mycobacterium tuberculosis* infection through an extracellular cytokine-like pathway (Bogunovic et al., 2012; Kimmey et al., 2017). Mx1 has noteworthy antiviral effects on hematopoietic cells, alongside its recognized antiviral activity on nonhematopoietic cells (Spitaels et al., 2019; Gough et al., 2022). OASL induces amyloid fibrillation in RIPK3, promoting virus-induced necrosis (Lee et al., 2023). OASL is strongly induced upon viral infection to enhance the antiviral IFN response (Leisching et al., 2017; Ghosh et al., 2019). The association between OASL and TB infection has also been validated, and OASL plays a central role in COVID-19 immunopathogenesis (Zhang et al., 2019; Hasankhani et al., 2021; Yi et al., 2021). RSAD2 is the direct suppressor of viral replication and facilitates TLR9- and TLR7-mediated production of IFN-α (Mantovani et al., 2022). The adaptive behavior of NK cells during viral infection is facilitated through STAT1-mediated epigenetic control of RSAD2 (Wiedemann et al., 2020). GBP1 was found to be one of the potential biomarkers of active TB, and its expression was negatively correlated with lymphocyte activity but positively correlated with myeloid and inflammatory cell activity (Perumal et al., 2020; Chen et al., 2022). In severe conditions of COVID-19, there is a high abundance of CXCL10+ and CCL2+ inflammatory macrophages that heavily express the GBP1 inflammatory gene (Zhang et al., 2020). OAS1 acts as a negative regulator of the expression of chemokines and interferon-responsive genes in human macrophages (Lee et al., 2019). Reduced OAS1 expression due to a common haplotype is proven to be related to the severity of COVID-19 (Banday et al., 2022). Genetically regulated loss of OAS1 expression contributes to impaired spontaneous clearance of SARS-CoV-2 and an increased risk of hospitalization for COVID-19 (Banday et al., 2021; Huffman et al., 2022). Compared to normal tissues, IFI6 is markedly upregulated in white blood cells from COVID-19 patients and nasopharyngeal tissues infected with SARS-COV-2 and is associated with antiviral immune modulation and clinical progression (Dong et al., 2022; Sun et al., 2023; Villamayor et al., 2023). The antiviral activity displayed by HERC5 makes them promising drug targets for the development of novel antiviral therapeutics that can augment the host antiviral response (Jacquet et al., 2020; Mathieu et al., 2021).

TFs and miRNAs that act as upstream regulators of these hub genes have also been discovered to better understand the pathological basis of these disease states. Transcription factor GATA2 is associated with hematopoietic dysfunction in severe COVID-19 patients (Wang et al., 2021). Downregulation of GATA2, involved in lymphocyte commitment, is also found in TB (Li et al., 2023). The phosphorylation of STAT1 was reportedly strengthened in severe COVID-19 cases that failed to induce transcription of interferon-stimulated response elements (ISRE) by unbalanced JAK/STAT signaling (Rincon-Arevalo et al., 2022). MiR-26b-5p targeted cyclooxygenase 2 (COX2), leading to a decrease in its expression. Consequently, there were considerable reductions in the levels of proinflammatory mediators such as prostaglandin E2 (PGE2), TNF-α, and IL-6 in retinal artery and human dermal microvascular endothelial cells (HMEC-1). This resulted in an effective inhibition of inflammation (Jiang et al., 2020). MiR-26b-5p was predicted to be regulated by spike, ACE, and histone deacetylation (HDAC) pathways in COVID-19 (Teodori et al., 2020). Although many previous studies have suggested that these TFs and miRNAs may have potential therapeutic effects, these analytical results require further experiments to confirm their effectiveness and authenticity.

Drugs tending to regulate the hub genes were identified from the DSigDB database. The hub genes, which act as regulators of the common pathogenic processes of both diseases and can lead to the simultaneous administration of antiviral and anti-TB drugs, have the potential to provide significant clinical benefit to this patient population. Some of the drugs identified in this study are proposed by different authors as therapeutics for the treatment of COVID-19 or TB, for example, (1) suloctidil is found to be one of the candidate drugs between COVID-19 and idiopathic pulmonary fibrosis (Chen et al., 2022); (2) acetohexamide is a nucleotide-binding domain (NBD)-binding drug that can be used against COVID-19 by preventing replication and viral attachment to the cell surface binding immunoglobin protein (csBiP) (Zhang et al., 2021); (3) The use of prochlorperazine has been shown to decrease the replication ability of SARS-CoV-2, which is linked to the ACE2 receptor. Also, prochlorperazine is found significant inhibition in lung pathology and lung viral load of SARS-CoV-2-challenged hamsters. Prochlorperazine can bind to G-quadruplexes (G4s), a secondary structure in nucleic acids that is known to impact numerous cellular processes, including viral replication and transcription. This binding can impede SARS-CoV-2 reverse transcription and ultimately reduce the lung viral load (Roy et al., 2023). (4) The application of chlorophyllin and molnupiravir as a specific antiviral drug for SARS-CoV-2 can diminish the detrimental genetic alterations and host cell harm caused by molnupiravir while enhancing the therapeutic effectiveness (Clark et al., 2022).

While the genes we have identified offer fresh perspectives on the creation of potential therapeutic targets for COVID-19 and TB, the validation of these gene targets and drugs requires continued investigation through experimental studies like cellular and animal models. Also, information and methodological biases preclude the full reproduction of potential genetic links using computational biology techniques. Additionally, translating experimental results into clinical applications remains a significant challenge. These are the main limitations of our current study and the focus of our future research.

## Conclusion

To help get insights into the common pathogenetic processes between the SARS-CoV2 infection and TB, we utilized transcriptomic data analysis to determine the shared pathways and biomarkers in TB and COVID-19. There are 96 common DEGs of TB and COVID-19 identified by bioinformatics tools. GO terms and signaling pathway enrichment analysis revealed that 96 common DEGs were mainly involved in the regulation of viral genome replication and immune-related pathways. Moreover, through the PPI analysis of DEGs, the top 10 hub genes were extracted, including IFI44L, ISG15, MX1, IFI44, OASL, RSAD2, GBP1, OAS1, IFI6, and HERC5, which may be a therapeutic target for COVID-19 and help to find drug molecules and drug-target interactions. Also, gene–TF and gene–miRNA association were examined to gain a deeper understanding of COVID-19 progression. In addition, several potential drugs were listed for COVID-19 patients with TB treatment, including suloctidil, prenylamine, acetohexamide, terfenadine, prochlorperazine, 3′-azido-3′-deoxythymidine, chlorophyllin, etoposide, clioquinol, and propofol. We hope these findings may provide key perspectives for developing novel and effective medications to combat COVID-19 and TB.

## Data availability statement

The original contributions presented in the study are included in the article/**Supplementary Material**. Further inquiries can be directed to the corresponding authors.

## Author contributions

TH: Data curation, Methodology, Supervision, Writing – original draft. JH: Investigation, Supervision, Writing – original draft. XZ: Conceptualization, Project administration, Writing – review & editing. HP: Visualization, Writing – review & editing. FH: Investigation, Writing – review & editing. AD: Project administration, Writing – review & editing. BY: Conceptualization, Writing – original draft. NJ: Conceptualization, Writing – original draft. XL: Project administration, Writing – review & editing. KY: Methodology, Writing – review & editing. ZW: Investigation, Project administration, Writing – original draft.

## Supplementary material

Supplementary Table 1 The baseline characteristic of sample in COVID-19 (GSE196822).

Supplementary Table 2 The baseline characteristic of sample in TB (GSE126614).

Supplementary Table 3 The differential analysis matrix of COVID-19.

Supplementary Table 4 The differential analysis matrix of TB.

## References

[B1] Aggarwal A. N. Agarwal R. Dhooria S. Prasad K. T. Sehgal I. S. Muthu V. (2021). Active pulmonary tuberculosis and coronavirus disease 2019: A systematic review and meta-analysis. PloS One 16 (10), e0259006. doi: 10.1371/journal.pone.0259006 34673822 PMC8530351

[B2] Al-Mustanjid M. Mahmud S. M. H. Royel M. R. I. Rahman M. H. Islam T. Rahman M. R.. (2020). Detection of molecular signatures and pathways shared in inflammatory bowel disease and colorectal cancer: A bioinformatics and systems biology approach. Genomics 112 (5), 3416–3426. doi: 10.1016/j.ygeno.2020.06.001 32535071

[B3] Banday A. R. Stanifer M. L. Florez-Vargas O. Onabajo O. O. Papenberg B. W. Zahoor M. A.. (2022). Genetic regulation of OAS1 nonsense-mediated decay underlies association with COVID-19 hospitalization in patients of European and African ancestries. Nat. Genet. 54 (8), 1103–1116. doi: 10.1038/s41588-022-01113-z 35835913 PMC9355882

[B4] Bardou P. Mariette J. Escudié F. Djemiel C. Klopp C. (2014). jvenn: an interactive Venn diagram viewer. BMC Bioinf. 15 (1), 1–7. doi: 10.1186/1471-2105-15-293 PMC4261873 25176396

[B5] Barrett T. Wilhite S. E. Ledoux P. Evangelista C. Kim I. F. Tomashevsky M.. (2013). NCBI GEO: archive for functional genomics data sets–update. Nucleic Acids Res. 41 (Database issue), D991–D995. doi: 10.1093/nar/gks1193 23193258 PMC3531084

[B6] Behr M. A. Kaufmann E. Duffin J. Edelstein P. H. Ramakrishnan L. (2021). Latent Tuberculosis: Two Centuries of Confusion. Am. J. Respir. Crit. Care Med. 204(2), 142–148. doi: 10.1164/rccm.202011-4239PP 33761302 PMC8650795

[B7] Benn C. S. Netea M. G. Selin L. K. Aaby P. (2013). A small jab - a big effect: nonspecific immunomodulation by vaccines. Trends Immunol. 34 (9), 431–439. doi: 10.1016/j.it.2013.04.004 23680130

[B8] Bhat M. Y. Solanki H. S. Advani J. Khan A. A. Keshava Prasad T. S. Gowda H.. (2018). Comprehensive network map of interferon gamma signaling. J. Cell Commun. Signal 12 (4), 745–751. doi: 10.1007/s12079-018-0486-y 30191398 PMC6235777

[B9] Bogunovic D. Byun M. Durfee L. A. Abhyankar A. Sanal O. Mansouri D.. (2012). Mycobacterial disease and impaired IFN-γ immunity in humans with inherited ISG15 deficiency. Science 337 (6102), 1684–1688. doi: 10.1126/science.1224026 22859821 PMC3507439

[B10] Boutin S. Hildebrand D. Boulant S. Kreuter M. Rüter J. Pallerla S. R.. (2021). Host factors facilitating SARS-CoV-2 virus infection and replication in the lungs. Cell Mol. Life Sci. 78 (16), 5953–5976. doi: 10.1007/s00018-021-03889-5 34223911 PMC8256233

[B11] Busse D. C. Habgood-Coote D. Clare S. Brandt C. Bassano I. Kaforou M.. (2020). Interferon-induced protein 44 and interferon-induced protein 44-like restrict replication of respiratory syncytial virus. J. Virol. 94 (18), e00297-20. doi: 10.1128/JVI.00297-20 32611756 PMC7459546

[B12] Callaway E. (2021). Fast-spreading COVID variant can elude immune responses. Nature 589 (7843), 500–501. doi: 10.1038/d41586-021-00121-z 33479534

[B13] Chakaya J. Khan M. Ntoumi F. Aklillu E. Fatima R. Mwaba P.. (2021). Global Tuberculosis Report 2020–Reflections on the Global TB burden, treatment and prevention efforts. Int. J. Infect. Dis. 113, S7–S12. doi: 10.1016/j.ijid.2021.02.107 33716195 PMC8433257

[B14] Chan J. F. Kok K. H. Zhu Z. Chu H. To K. K. Yuan S. Yuen K. Y. (2020). Genomic characterization of the 2019 novel human-pathogenic coronavirus isolated from a patient with atypical pneumonia after visiting Wuhan. Emerg. Microbes Infect. 9 (1), 221–236. doi: 10.1080/22221751.2020.1719902 31987001 PMC7067204

[B15] Chen L. Hua J. He X. (2022). Coexpression network analysis-based identification of critical genes differentiating between latent and active tuberculosis. Dis. Markers 2022, 2090560. doi: 10.1155/2022/2090560 36411825 PMC9674975

[B16] Chen Y. Guo Y. Pan Y.. (2020). Structure analysis of the receptor binding of 2019-nCoV. Biochem. Biophys. Res. Commun. 525 (1), 135–140. doi: 10.1016/j.bbrc.2020.02.071 32081428 PMC7092824

[B17] Chen Q. Xia S. Sui H. Shi X. Huang B. Wang T. (2022). Identification of hub genes associated with COVID-19 and idiopathic pu lmonary fibrosis by integrated bioinformatics analysis. PloS One 17 (1), e0262737. doi: 10.1371/journal.pone.0262737 35045126 PMC8769324

[B18] Clark N. F. Taylor-Robinson A. W. Heimann K. (2022). Could chlorophyllins improve the safety profile of beta-d-N4-hydroxycy tidine versus N-hydroxycytidine, the active ingredient of the SARS-CoV -2 antiviral molnupiravir? Ther. Adv. Drug Saf. 13. 20420986221107753. doi: 10.1177/20420986221107753 PMC9309465 35898799

[B19] Dediego M. L. Martinez-Sobrido L. Topham D. J. (2019). Novel functions of IFI44L as a feedback regulator of host antiviral responses. J. Virol. 93 (21), e01159-19. doi: 10.1128/JVI.01159-19 31434731 PMC6803278

[B20] Del Rosario R. C. H. Poschmann J. Lim C. Cheng C. Y. Kumar P. Riou C.. (2022). Author Correction: Histone acetylome-wide associations in immune cells from individuals with active Mycobacterium tuberculosis infection. Nat. Microbiol. 7 (11), 1943. doi: 10.1038/s41564-022-01236-3 36056160 PMC9613471

[B21] Deng S. Shen S. Liu K. El-Ashram S. Alouffi A. Cenci-Goga B. T.. (2023). Integrated bioinformatic analyses investigate macrophage-M1-related biomarkers and tuberculosis therapeutic drugs. Front. Genet. 14, 1041892. doi: 10.3389/fgene.2023.1041892 36845395 PMC9945105

[B22] Deshpande S. S. Joshi A. R. Shah A. (2020). Aftermath of pulmonary tuberculosis: computed tomography assessment. Pol. J. Radiol. 85, e144–e154. doi: 10.5114/pjr.2020.93714 32322321 PMC7172931

[B23] Dong Z. Yan Q. Cao W. Liu Z. Wang X. (2022). Identification of key molecules in COVID-19 patients significantly correlated with clinical outcomes by analyzing transcriptomic data. Front. Immunol. 13, 930866. doi: 10.3389/fimmu.2022.930866 36072597 PMC9441550

[B24] Fonseca C. A. Jr. Zanetti G. Marchiori E. (2021). Pulmonary tuberculosis in a patient with COVID-19 pneumonia. Rev. Soc. Bras. Med. Trop. 54, e03142021. doi: 10.1590/0037-8682-0314-2021 34231776 PMC8253577

[B25] Fornes O. Castro-Mondragon J. A. Khan A. van der Lee R. Zhang X. Richmond P. A.. (2020). JASPAR 2020: update of the open-access database of transcription factor binding profiles. Nucleic Acids Res. 48 (D1), D87–D92. doi: 10.1093/nar/gkz1001 31701148 PMC7145627

[B26] Gao Y. Liu M. Chen Y. Shi S. Geng J. Tian J. (2021). Association between tuberculosis and COVID-19 severity and mortality: A rapid systematic review and meta-analysis. J. Med. Virol. 93 (1), 194–196. doi: 10.1002/jmv.26311 32687228 PMC7405273

[B27] Geerling E. Pinski A. N. Stone T. E. DiPaolo R. J. Zulu M. Z. Maroney K. J.. (2022). Roles of antiviral sensing and type I interferon signaling in the restriction of SARS-CoV-2 replication. iScience 25 (1), 103553. doi: 10.1016/j.isci.2021.103553 34877479 PMC8639477

[B28] Gheblawi M. Wang K. Viveiros A. Nguyen Q. Zhong J.-C. Turner A. J.. (2020). Angiotensin-converting enzyme 2: SARS-CoV-2 receptor and regulator of the renin-angiotensin system: celebrating the 20th anniversary of the discovery of ACE2. Circ. Res. 126 (10), 1456–1474. doi: 10.1161/CIRCRESAHA.120.317015 32264791 PMC7188049

[B29] Ghosh A. Shao L. Sampath P. Zhao B. Patel N. V. Zhu J.. (2019). Oligoadenylate-Synthetase-Family Protein OASL Inhibits Activity of the DNA Sensor cGAS during DNA Virus Infection to Limit Interferon Production. Immunity 50 (1), 51–63.e5. doi: 10.1016/j.immuni.2018.12.013 30635239 PMC6342484

[B30] Gold I. M. Reis N. Glaser F. Glickman M. H. (2022). Coronaviral PLpro proteases and the immunomodulatory roles of conjugated versus free Interferon Stimulated Gene product-15 (ISG15). Semin. Cell Dev. Biol. 132, 16–26. doi: 10.1016/j.semcdb.2022.06.005 35764457 PMC9233553

[B31] Gough M. Singh D. K. Singh B. Kaushal D. Mehra S. (2022). System-wide identification of myeloid markers of TB disease and HIV-induced reactivation in the macaque model of Mtb infection and Mtb/SIV co-infection. Front. Immunol. 13, 777733. doi: 10.3389/fimmu.2022.777733 36275677 PMC9583676

[B32] Guan W. J. Ni Z. Y. Hu Y. Liang W. H. Ou C. Q. He J. X.. (2020). Clinical characteristics of coronavirus disease 2019 in China. N Engl. J. Med. 382 (18), 1708–1720. doi: 10.1056/NEJMoa2002032 32109013 PMC7092819

[B33] Hasankhani A. Bahrami A. Sheybani N. Aria B. Hemati B. Fatehi F. (2021). Differential co-expression network analysis reveals key hub-high traffic genes as potential therapeutic targets for COVID-19 pandemic. Front. Immunol. 12, 789317. doi: 10.3389/fimmu.2021.789317 34975885 PMC8714803

[B34] Hildebrand R. E. Chandrasekar S. S. Riel M. Touray B. J. B. Aschenbroich S. A. Talaat A. M. (2022). Superinfection with SARS-CoV-2 Has Deleterious Effects on Mycobacterium bovis BCG Immunity and Promotes Dissemination of Mycobacterium tuberculosis. Microbiol. Spectr. 10 (5), e0307522. doi: 10.1128/spectrum.03075-22 36200898 PMC9603897

[B35] Huang T. Jiang N. Song Y. Pan H. Du A. Yu B.. (2023a). Bioinformatics and system biology approach to identify the influences of SARS-CoV2 on metabolic unhealthy obese patients. Front. Mol. Biosci. 10, 1274463. doi: 10.3389/fmolb.2023.1274463 37877121 PMC10591333

[B36] Huang H.-Y. Lin Y-C-D Li J. Huang K.-Y. Shrestha S. Hong H.-C.. (2020). miRTarBase 2020: updates to the experimentally validated microRNA–target interaction database. Nucleic Acids Res. 48 (D1), D148–D154. doi: 10.1093/nar/gkz896 31647101 PMC7145596

[B37] Huang T. Yu B. Zhou X. Pan H. Du A. Bai J.. (2023a). Exploration of the link between COVID-19 and alcoholic hepatitis from the perspective of bioinformatics and systems biology. MedComm–Future Med. 2 (2), e42. doi: 10.1002/mef2.42

[B38] Huang T. Zheng D. Song Y. Pan H. Qiu G. Xiang Y.. (2023b). Demonstration of the impact of COVID-19 on metabolic associated fatty liver disease by bioinformatics and system biology approach. Medicine 102 (35), e34570. doi: 10.1097/MD.0000000000034570 37657050 PMC10476796

[B39] Huffman J. E. Butler-Laporte G. Khan A. Pairo-Castineira E. Drivas T. G. Peloso G. M.. (2022). Multi-ancestry fine mapping implicates OAS1 splicing in risk of severe COVID-19. Nat. Genet. 54 (2), 125–127. doi: 10.1038/s41588-021-00996-8 35027740 PMC8837537

[B40] Jacquet S. Pontier D. Etienne L. (2020). Rapid evolution of HERC6 and duplication of a chimeric HERC5/6 gene in rodents and bats suggest an overlooked role of HERCs in mammalian immunity. Front. Immunol. 11, 605270. doi: 10.3389/fimmu.2020.605270 33391270 PMC7775381

[B41] Jiang S. Chen Z. Lai W. Mai Q. Chen D. Sun S.. (2020). Decoction of heat-clearing, detoxifying and blood stasis removing relieves acute soft tissue injury via modulating miR-26b-5p/COX2 axis to inhibit inflammation. Biosci. Rep. 40 (12), BSR20201981. doi: 10.1042/BSR20201981 33270831 PMC7753743

[B42] Jiang H. Tsang L. Wang H. Liu C. (2021). IFI44L as a forward regulator enhancing host antituberculosis responses. J. Immunol. Res. 2021, 5599408. doi: 10.1155/2021/5599408 34722780 PMC8550841

[B43] Jin Y. Yang H. Ji W. Wu W. Chen S. Zhang W.. (2020). Virology, epidemiology, pathogenesis, and control of COVID-19. Viruses 12 (4), 372. doi: 10.3390/v12040372 32230900 PMC7232198

[B44] Kimmey J. M. Campbell J. A. Weiss L. A. Monte K. J. Lenschow D. J. Stallings C. L. (2017). The impact of ISGylation during Mycobacterium tuberculosis infection in mice. Microbes Infect. 19 (4-5), 249–258. doi: 10.1016/j.micinf.2016.12.006 28087453 PMC5403610

[B45] Kuleshov M. V. Jones M. R. Rouillard A. D. Fernandez N. F. Duan Q. Wang Z.. (2016). Enrichr: a comprehensive gene set enrichment analysis web server 2016 update. Nucleic Acids Res. 44 (W1), W90–W97. doi: 10.1093/nar/gkw377 27141961 PMC4987924

[B46] Kvam V. M. Liu P. Si Y. (2012). A comparison of statistical methods for detecting differentially expressed genes from RNA-seq data. Am. J. Bot. 99 (2), 248–256. doi: 10.3732/ajb.1100340 22268221

[B47] Lee S. A. Chang L. C. Jung W. Bowman J. W. Kim D. Chen W.. (2023). OASL phase condensation induces amyloid-like fibrillation of RIPK3 to promote virus-induced necroptosis. Nat. Cell Biol. 25 (1), 92–107. doi: 10.1038/s41556-022-01039-y 36604592 PMC9859756

[B48] Lee W. B. Choi W. Y. Lee D. H. Shim H. Kim-Ha J. Kim Y. J. (2019). OAS1 and OAS3 negatively regulate the expression of chemokines and interferon-responsive genes in human macrophages. BMB Rep. 52 (2), 133–138. doi: 10.5483/BMBRep.2019.52.2.129 30078389 PMC6443328

[B49] Leisching G. Wiid I. Baker B. (2017). The association of OASL and type I interferons in the pathogenesis and survival of intracellular replicating bacterial species. Front. Cell Infect. Microbiol. 7, 196. doi: 10.3389/fcimb.2017.00196 28580319 PMC5437694

[B50] Li Y. Liu Y. Duo M. Wu R. Jiang T. Li P.. (2022). Bioinformatic analysis and preliminary validation of potential therapeutic targets for COVID-19 infection in asthma patients. Cell Communication Signaling 20 (1), 1–12. doi: 10.1186/s12964-022-01010-2 36575422 PMC9793391

[B51] Li F. Ma Y. Li X. Zhang D. Han J. Tan D.. (2023). Severe persistent mycobacteria antigen stimulation causes lymphopenia through impairing hematopoiesis. Front. Cell Infect. Microbiol. 13, 1079774. doi: 10.3389/fcimb.2023.1079774 36743311 PMC9889370

[B52] Li Y. Qi J. Yang J. (2021a). RTP4 is a novel prognosis-related hub gene in cutaneous melanoma. Hereditas 158 (1), 22. doi: 10.1186/s41065-021-00183-z 34154655 PMC8215788

[B53] Li Y. Zhang J. Wang C. Qiao W. Li Y. Tan J. (2021b). IFI44L expression is regulated by IRF-1 and HIV-1. FEBS Open Bio 11 (1), 105–113. doi: 10.1002/2211-5463.13030 PMC7780093 33159419

[B54] Love M. I. Huber W. Anders S. (2014). Moderated estimation of fold change and dispersion for RNA-seq data with DESeq2. Genome Biol. 15 (12), 1–21. doi: 10.1186/s13059-014-0550-8 PMC4302049 25516281

[B55] Lukhele S. Boukhaled G. M. Brooks D. G. (2019). Type I interferon signaling, regulation and gene stimulation in chronic virus infection. Semin. Immunol. 43, 101277. doi: 10.1016/j.smim.2019.05.001 31155227 PMC8029807

[B56] Ma H. He Z. Chen J. Zhang X. Song P. (2021). Identifying of biomarkers associated with gastric cancer based on 11 topological analysis methods of CytoHubba. Sci. Rep. 11 (1), 1–11. doi: 10.1038/s41598-020-79235-9 33446695 PMC7809423

[B57] Mantovani S. Daga S. Fallerini C. Baldassarri M. Benetti E. Picchiotti N.. (2022). Rare variants in Toll-like receptor 7 results in functional impairment and downregulation of cytokine-mediated signaling in COVID-19 patients. Genes Immun. 23 (1), 51–56. doi: 10.1038/s41435-021-00157-1 34952932 PMC8703210

[B58] Mathieu N. A. Paparisto E. Barr S. D. Spratt D. E. (2021). HERC5 and the ISGylation pathway: critical modulators of the antiviral immune response. Viruses 13 (6), 1102. doi: 10.3390/v13061102 34207696 PMC8228270

[B59] Mousquer G. T. Peres A. Fiegenbaum M. (2021). Pathology of TB/COVID-19 Co-Infection: The phantom menace. Tuberculosis (Edinb) 126, 102020. doi: 10.1016/j.tube.2020.102020 33246269 PMC7669479

[B60] Munnur D. Banducci-Karp A. Sanyal S. (2022). ISG15 driven cellular responses to virus infection. Biochem. Soc. Trans. 50 (6), 1837–1846. doi: 10.1042/BST20220839 36416643 PMC9788361

[B61] Perlman S. Netland J. (2009). Coronaviruses post-SARS: update on replication and pathogenesis. Nat. Rev. Microbiol. 7 (6), 439–450. doi: 10.1038/nrmicro2147 19430490 PMC2830095

[B62] Perumal P. Abdullatif M. B. Garlant H. N. Honeyborne I. Lipman M. McHugh T. D.. (2020). Validation of differentially expressed immune biomarkers in latent and active tuberculosis by real-time PCR. Front. Immunol. 11, 612564. doi: 10.3389/fimmu.2020.612564 33841389 PMC8029985

[B63] Pollard C. A. Morran M. P. Nestor-Kalinoski A. L. (2020). The COVID-19 pandemic: a global health crisis. Physiol. Genomics 52 (11), 549–557. doi: 10.1152/physiolgenomics.00089.2020 32991251 PMC7686876

[B64] Ragab D. Salah Eldin H. Taeimah M. Khattab R. Salem R. (2020). The COVID-19 cytokine storm; what we know so far. Front. Immunol. 11, 1446. doi: 10.3389/fimmu.2020.01446 32612617 PMC7308649

[B65] Rahman S. Gudetta B. Fink J. Granath A. Ashenafi S. Aseffa A.. (2009). Compartmentalization of immune responses in human tuberculosis: few CD8+ effector T cells but elevated levels of FoxP3+ regulatory t cells in the granulomatous lesions. Am. J. Pathol. 174 (6), 2211–2224. doi: 10.2353/ajpath.2009.080941 19435796 PMC2684186

[B66] Raso M. C. Djoric N. Walser F. Hess S. Schmid F. M. Burger S.. (2020). Interferon-stimulated gene 15 accelerates replication fork progression inducing chromosomal breakage. J. Cell Biol. 219 (8), e202002175. doi: 10.1083/jcb.202002175 32597933 PMC7401800

[B67] Rincon-Arevalo H. Aue A. Ritter J. Szelinski F. Khadzhynov D. Zickler D.. (2022). Altered increase in STAT1 expression and phosphorylation in severe COVID-19. Eur. J. Immunol. 52 (1), 138–148. doi: 10.1002/eji.202149575 34676541 PMC8646801

[B68] Rivas M. N. Ebinger J. E. Wu M. Sun N. Braun J. Sobhani K.. (2021). BCG vaccination history associates with decreased SARS-CoV-2 seroprevalence across a diverse cohort of health care workers. J. Clin. Invest. 131 (2), e145157. doi: 10.1172/JCI145157 33211672 PMC7810479

[B69] Roy S. S. Sharma S. Rizvi Z. A. Sinha D. Gupta D. Rophina M.. (2023). G4-binding drugs, chlorpromazine and prochlorperazine, repurposed agai nst COVID-19 infection in hamsters. Front. Mol. Biosci. 10, 1133123. doi: 10.3389/fmolb.2023.1133123 37006620 PMC10061221

[B70] Rustad T. R. Minch K. J. Ma S. Winkler J. K. Hobbs S. Hickey M.. (2014). Mapping and manipulating the Mycobacterium tuberculosis transcriptome using a transcription factor overexpression-derived regulatory network. Genome Biol. 15 (11), 1–11. doi: 10.1186/s13059-014-0502-3 PMC4249609 25380655

[B71] Shi H. Han X. Jiang N. Cao Y. Alwalid O. Gu J.. (2020). Radiological findings from 81 patients with COVID-19 pneumonia in Wuhan, China: a descriptive study. Lancet Infect. Dis. 20 (4), 425–434. doi: 10.1016/S1473-3099(20)30086-4 32105637 PMC7159053

[B72] Smoot M. E. Ono K. Ruscheinski J. Wang P. L. Ideker T. (2011). Cytoscape 2.8: new features for data integration and network visualization. Bioinformatics 27 (3), 431–432. doi: 10.1093/bioinformatics/btq675 21149340 PMC3031041

[B73] Song Y. Huang T. Pan H. Du A. Wu T. Lan J.. (2023). The influence of COVID-19 on colorectal cancer was investigated using bioinformatics and systems biology techniques. Front. Med. 10. doi: 10.3389/fmed.2023.1169562 PMC10348756 37457582

[B74] Spinato G. Fabbris C. Polesel J. Cazzador D. Borsetto D. Hopkins C.. (2020). Alterations in smell or taste in mildly symptomatic outpatients with SARS-coV-2 infection. JAMA 323 (20), 2089–2090. doi: 10.1001/jama.2020.6771 32320008 PMC7177631

[B75] Spitaels J. Van Hoecke L. Roose K. Kochs G. Saelens X. (2019). Mx1 in hematopoietic cells protects against thogoto virus infection. J. Virol. 93 (15), e00193-19. doi: 10.1128/JVI.00193-19 31092574 PMC6639282

[B76] Subramanian A. Tamayo P. Mootha V. K. Mukherjee S. Ebert B. L. Gillette M. A.. (2005). Gene set enrichment analysis: a knowledge-based approach for interpreting genome-wide expression profiles. Proc. Natl. Acad. Sci. 102 (43), 15545–15550. doi: 10.1073/pnas.0506580102 16199517 PMC1239896

[B77] Sun Z. Ke L. Zhao Q. Qu J. Hu Y. Gao H.. (2023). The use of bioinformatics methods to identify the effects of SARS-CoV-2 and influenza viruses on the regulation of gene expression in patients. Front. Immunol. 14, 1098688. doi: 10.3389/fimmu.2023.1098688 36911695 PMC9992716

[B78] Szklarczyk D. Gable A. L. Lyon D. Junge A. Wyder S. Huerta-Cepas J.. (2019). STRING v11: protein-protein association networks with increased coverage, supporting functional discovery in genome-wide experimental datasets. Nucleic Acids Res. 47 (D1), D607–D613. doi: 10.1093/nar/gky1131 30476243 PMC6323986

[B79] Tadolini M. Codecasa L. R. García-García J. M. Blanc F. X. Borisov S. Alffenaar J. W.. (2020). Active tuberculosis, sequelae and COVID-19 co-infection: first cohort of 49 cases. Eur. Respir. J. 56 (1), 2001398. doi: 10.1183/13993003.01398-2020 32457198 PMC7251245

[B80] Tan L. Wang Q. Zhang D. Ding J. Huang Q. Tang Y. Q.. (2020). Lymphopenia predicts disease severity of COVID-19: a descriptive and predictive study. Signal Transduct Target Ther. 5 (1), 33. doi: 10.1038/s41392-020-0148-4 32296069 PMC7100419

[B81] Teodori L. Sestili P. Madiai V. Coppari S. Fraternale D. Rocchi M. B.L.. (2020). MicroRNAs bioinformatics analyses identifying HDAC pathway as a putative target for existing anti-COVID-19 therapeutics. Front. Pharmacol. 11, 582003. doi: 10.3389/fphar.2020.582003 33363465 PMC7753186

[B82] Trugilho M. R. O. Azevedo-Quintanilha I. G. Gesto J. S. M. Moraes E. C. S. Mandacaru S. C. Campos M. M.. (2022). Platelet proteome reveals features of cell death, antiviral response and viral replication in covid-19. Cell Death Discovery 8 (1), 324. doi: 10.1038/s41420-022-01122-1 35842415 PMC9287722

[B83] Umakanthan S. Sahu P. Ranade A. V. Bukelo M. M. Rao J. S. Abrahao-Machado L. F.. (2020). Origin, transmission, diagnosis and management of coronavirus disease 2019 (COVID-19). Postgraduate Med. J. 96 (1142), 753–758. doi: 10.1136/postgradmedj-2020-138234 PMC10016932 32563999

[B84] Villamayor L. Rivero V. López-García D. Topham D. J. Martínez-Sobrido L. Nogales A.. (2023). Interferon alpha inducible protein 6 is a negative regulator of innate immune responses by modulating RIG-I activation. Front. Immunol. 14, 1105309. doi: 10.3389/fimmu.2023.1105309 36793726 PMC9923010

[B85] Visca D. Ong C. W. M. Tiberi S. Centis R. D'Ambrosio L. Chen B.. (2021). Tuberculosis and COVID-19 interaction: A review of biological, clinical and public health effects. Pulmonology 27 (2), 151–165. doi: 10.1016/j.pulmoe.2020.12.012 33547029 PMC7825946

[B86] Wang X. Wen Y. Xie X. Liu Y. Tan X. Cai Q.. (2021). Dysregulated hematopoiesis in bone marrow marks severe COVID-19. Cell Discovery 7 (1), 60. doi: 10.1038/s41421-021-00296-9 34349096 PMC8335717

[B87] Wiedemann G. M. Geary C. D. Lau C. M. Sun J. C. (2020). Cutting edge: STAT1-mediated epigenetic control of rsad2 promotes clonal expansion of antiviral NK cells. J. Immunol. 205 (1), 21–25. doi: 10.4049/jimmunol.2000086 32461239 PMC7314649

[B88] Yi F. Hu J. Zhu X. Wang Y. Yu Q. Deng J.. (2021). Transcriptional profiling of human peripheral blood mononuclear cells stimulated by mycobacterium tuberculosis PPE57 identifies characteristic genes associated with type I interferon signaling. Front. Cell Infect. Microbiol. 11, 716809. doi: 10.3389/fcimb.2021.716809 34490145 PMC8416891

[B89] Yoo M. Shin J. Kim J. Ryall K. A. Lee K. Lee S.. (2015). DSigDB: drug signatures database for gene set analysis. Bioinformatics 31 (18), 3069–3071. doi: 10.1093/bioinformatics/btv313 25990557 PMC4668778

[B90] Zhai W. Wu F. Zhang Y. Fu Y. Liu Z. (2019). The immune escape mechanisms of mycobacterium tuberculosis. Int. J. Mol. Sci. 20 (2), 340. doi: 10.3390/ijms20020340 30650615 PMC6359177

[B91] Zhang Y. Greer R. A. Song Y. Praveen H. Song Y. (2021). In silico identification of available drugs targeting cell surface BiP to disrupt SARS-CoV-2 binding and replication: Drug repurposing approach. Eur. J. Pharm. Sci. 160, 105771. doi: 10.1016/j.ejps.2021.105771 33617948 PMC7894100

[B92] Zhang Y. W. Lin Y. Yu H. Y. Tian R. N. Li F. (2019). Characteristic genes in THP−1 derived macrophages infected with Mycobacterium tuberculosis H37Rv strain identified by integrating bioinformatics methods. Int. J. Mol. Med. 44 (4), 1243–1254. doi: 10.3892/ijmm.2019.4293 31364746 PMC6713430

[B93] Zhang F. Mears J. R. Shakib L. Beynor J. I. Shanaj S. Korsunsky I.. (2021). IFN- γ and TNF- α drive a CXCL10 + CCL2 + macrophage phenotype expanded in severe COVID-19 and other diseases with tissue inflammation. Genome Med 13(1), 64. doi: 10.1186/s13073-021-00881-3 33879239 PMC8057009

[B94] Zheng Q. Wang D. Lin R. Lv Q. Wang W. (2022). IFI44 is an immune evasion biomarker for SARS-CoV-2 and Staphylococcus aureus infection in patients with RA. Front. Immunol. 13, 1013322. doi: 10.3389/fimmu.2022.1013322 36189314 PMC9520788

[B95] Zhou G. Soufan O. Ewald J. Hancock R. E. Basu N. Xia J. (2019). NetworkAnalyst 3.0: a visual analytics platform for comprehensive gene expression profiling and meta-analysis. Nucleic Acids Res. 47 (W1), W234–W241. doi: 10.1093/nar/gkz240 30931480 PMC6602507

[B96] Ziegler C. G. K. Allon S. J. Nyquist S. K. Mbano I. M. Miao V. N. Tzouanas C. N.. (2020). SARS-coV-2 receptor ACE2 is an interferon-stimulated gene in human airway epithelial cells and is detected in specific cell subsets across tissues. Cell 181 (5), 1016–1035 e19. doi: 10.1016/j.cell.2020.04.035 32413319 PMC7252096

[B97] Zou X. Chen K. Zou J. Han P. Hao J. Han Z. (2020). Single-cell RNA-seq data analysis on the receptor ACE2 expression reveals the potential risk of different human organs vulnerable to 2019-nCoV infection. Front. Med. 14 (2), 185–192. doi: 10.1007/s11684-020-0754-0 32170560 PMC7088738

