# Supplementary material for: Discovering common pathogenetic processes between COVID-19 and tuberculosis by bioinformatics and system biology approach
Source: Front Cell Infect Microbiol. 2023 Dec 15;13:1280223. doi: 10.3389/fcimb.2023.1280223 (PMC10757339; doi:10.3389/fcimb.2023.1280223)
Supplement: Supplementary Table 1 — The baseline characteristic of sample in COVID-19 (GSE196822). [file Table_1.docx]

**Supplementary Table 1.** The baseline characteristic of sample in COVID-19 (GSE196822).

| Category | Healthy Control (n=9) | Asymptomatic (n=8) | Mild (n=9) | Moderate (n=10) | Severe (n=7) |
| --- | --- | --- | --- | --- | --- |
| Age, years | 31.4±13.6 | 35.4±25.6 | 50.7±34.3 | 52.7±17.3 | 62.1±17.9 |
| Sex |  |  |  |  |  |
| Male | 5 (55.6%) | 1 (12.5%) | 5 (55.6%) | 5 (50.0%) | 2 (28.6%) |
| Female | 4 (44.4%) | 7 (87.5%) | 4 (44.4%) | 5 (50.0%) | 5 (71.4%) |

**Supplementary Table 2.** The baseline characteristic of sample in TB (GSE126614).

| Category | Control (n=19) | TB (n=20) |
| --- | --- | --- |
| Age, years | 37.1±26.9 | 45.2±22.2 |
| Sex |  |  |
| Male | 14 (73.7%) | 12 (60.0%) |
| Female | 5 (26.3%) | 8 (40.0%) |
